# Structural polymorphism of amyloid fibrils in ATTR amyloidosis revealed by cryo-electron microscopy

**DOI:** 10.1038/s41467-024-44820-3

**Published:** 2024-01-17

**Authors:** Binh An Nguyen, Virender Singh, Shumaila Afrin, Anna Yakubovska, Lanie Wang, Yasmin Ahmed, Rose Pedretti, Maria del Carmen Fernandez-Ramirez, Preeti Singh, Maja Pękała, Luis O. Cabrera Hernandez, Siddharth Kumar, Andrew Lemoff, Roman Gonzalez-Prieto, Michael R. Sawaya, David S. Eisenberg, Merrill Douglas Benson, Lorena Saelices

**Affiliations:** 1 Center for Alzheimer’s and Neurodegenerative Diseases, University of Texas Southwestern Medical Center (UTSW), Dallas, TX USA; 2 Department of Biophysics, University of Texas Southwestern Medical Center (UTSW), Dallas, TX USA; 3 Peter O’Donnell Jr Brain Institute, University of Texas Southwestern Medical Center (UTSW), Dallas, TX USA; 4 Department of Biochemistry, University of Texas Southwestern Medical Center, Dallas, TX USA; 5 Andalusian Center for Molecular Biology and regenerative Medicine (CABIMER), Universidad de Sevilla-CSIC-Universidad-Pablo de Olavide, Departmento de Biología Celular, Facultad de Biología, Universidad de Sevilla, Sevilla, Spain; 6 Department of Biological Chemistry, University of California, Los Angeles, Howard Hughes Medical Institute, Los Angeles, CA USA; 7 Department of Pathology and Laboratory Medicine, Indiana University School of Medicine, Indianapolis, IN USA

**Keywords:** Cryoelectron microscopy, Protein aggregation

## Abstract

ATTR amyloidosis is caused by the deposition of transthyretin in the form of amyloid fibrils in virtually every organ of the body, including the heart. This systemic deposition leads to a phenotypic variability that has not been molecularly explained yet. In brain amyloid conditions, previous studies suggest an association between clinical phenotype and the molecular structures of their amyloid fibrils. Here we investigate whether there is such an association in ATTRv amyloidosis patients carrying the mutation I84S. Using cryo-electron microscopy, we determined the structures of cardiac fibrils extracted from three ATTR amyloidosis patients carrying the ATTRv-I84S mutation, associated with a consistent clinical phenotype. We found that in each ATTRv-I84S patient, the cardiac fibrils exhibited different local conformations, and these variations can co-exist within the same fibril. Our finding suggests that one amyloid disease may associate with multiple fibril structures in systemic amyloidoses, calling for further studies.

## Abstract (web summary)

In this work, the authors report Cryo-EM imaging revealing diversity in amyloid fibril structures among ATTR patients with the same genetic mutation I84S. Further study is warranted to grasp the implications in ATTR amyloidosis pathology.

## Introduction

Amyloidoses are a variable group of fatal disorders caused by the pathological accumulation of amyloid fibrils in affected organs^1^. In amyloid transthyretin (ATTR) amyloidosis, the amyloid fibrils are composed of the amyloidogenic form of the hormone transporter transthyretin^2^. ATTR self-assemblage caused by mutations in the *TTR* gene leads to variant ATTR (ATTRv) amyloidosis. Other unknown processes related to age lead to wild-type ATTR (ATTRwt) amyloidosis. The clinical presentation of ATTRv amyloidosis is unpredictable and variable, often manifesting between 25 and 65 years of age with polyneuropathy, autonomic neuropathy, gastrointestinal and eye involvement, carpal tunnel syndrome, spinal canal stenosis, and/or cardiomyopathy^3^. The clinical presentation of ATTRwt is more predictable and better characterized; it manifests later in life (> 60 years old) as cardiomyopathy, and mainly affects men^4^. The roots of the phenotypic variability of ATTR amyloidosis are yet unknown: is it connected to heterogeneity of fibril structures, or to characteristics of the surrounding tissue, or to other factors?

The technological advancements on cryo-electron microscopy (cryo-EM) have revolutionized the structural study of the variability, or polymorphism, of amyloid assemblies^5–10^. In particular, recent cryo-EM studies on tauopathies and synucleinopathies, a set of brain amyloid diseases, respectively, associated with the deposition of tau and α-synuclein fibrils, have found that each disease is associated with the same structure fold^11–16^. Yet, the same fibril fold can be shared by different diseases. An example is the case of Alzheimer’s disease, familial Danish dementia, and familial British dementia, which are tauopathies with different pathologies and yet the same fibril fold^17^. Nevertheless, in all these diseases, every patient studied with the same condition displayed the same fibril fold in their brain tissue. Several studies are now pursuing the structures of TMEM106B and amyloid-β fibrils to determine whether this correlation is found across all brain amyloid fibrils^5,18–20^. These structural studies on brain amyloid diseases have offered invaluable information about whether the amyloid structure is influenced by phenotype and vice versa. Further studies are warranted to include fibrils carrying disease-related mutations to assess the influence of genotype on fibril structure.

There are three previous cryo-EM studies on fibrils from ATTR amyloidosis patients, and they show the formation of distinct fibril polymorphs in two different organs: the heart^21,22^ and the vitreous humor^23^. Cardiac fibrils extracted from patients carrying the wild-type form of transthyretin and the ATTRv-V30M mutation share similar folds, but vitreous humor fibrils from an independent ATTRv-V30M patient appear structurally different. In the present work, we use cryo-EM to determine the molecular structures of fibrils extracted from the heart of three ATTRv-I84S (*pATTRv-I104S) patients. ATTRv-I84S amyloidosis is characterized by a consistent clinical presentation that starts with carpal tunnel, ocular deposition, and polyneuropathy, and ends with cardiomyopathy that leads to death^24^. We show that ATTRv-I84S amyloidosis patients present structural variations in their cardiac fibrils that are specific to the individual. Moreover, we found that ATTR fibril structures from each patient exhibited local structural variations, co-existing within the same fibril. Our study discusses the source and effects of fibril polymorphism in ATTR amyloidosis and whether there is a potential association between phenotype and fibril structure in systemic amyloidoses.

## Results

### Extraction and cryo-EM

We obtained freshly frozen or lyophilized cardiac tissue samples from three ATTRv-I84S patients from the same kindred. To extract ATTR fibrils, we used a purification method adapted from the one described by Schmidt et al. (Supplementary Fig. 1)^21^. Briefly, we minced and washed a small cardiac specimen with a tris-calcium buffer and incubated the insoluble material with collagenase overnight. Finally, we incubated the samples with EDTA and used ice water to extract amyloid into the soluble fraction. Pure ATTR fibrils from cardiac tissues eluted after one or two rounds of washing and centrifugation with ice water (Supplementary Fig. 1). We verified the presence of fibrils, evaluated the overall sample cleanliness and their suitability for cryo-EM structure determination using transmission electron microscopy with negative staining (Supplementary Fig. 2a). We also established the amyloid nature of the extracts by analyzing their capacity to seed TTR aggregation in vitro (Supplementary Fig. 2b). We typed the samples using an antibody that recognizes C-terminal fragments of transthyretin, which are only present in ATTR amyloidosis type A, in contrast to type B fibrils that contain uncleaved transthyretin (Supplementary Fig. 2c)^25^. After purification and confirmation, we screened and optimized samples for cryo-EM imaging and data collection (Supplementary Fig. 2d). In addition, using mass spectrometry, we showed that all fibril samples contain both wild-type and mutant transthyretin, confirming that all patients included in this study are heterozygous for the ATTRv-I84S mutation (Supplementary Tables 1 and 2 and Supplementary Fig. 3).

### ATTRv-I84S fibrils are structurally polymorphic

**Fig. 1:**
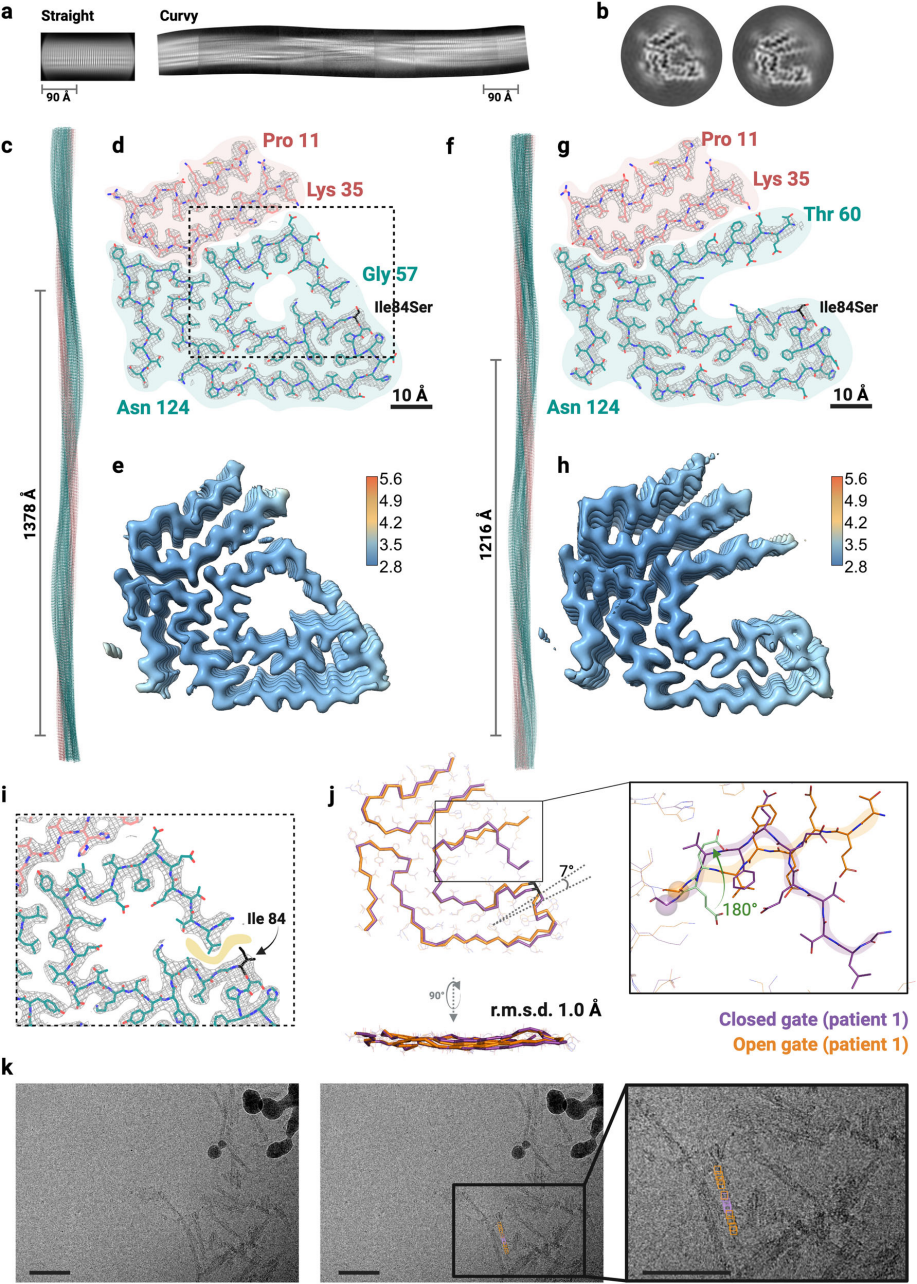
Cryo-EM structure of cardiac fibrils from ATTRv-I84S patient 1. **a** Representative 2D class averages of straight fibrils (left) and curvy fibrils after stitching (right). **b** 3D class averages of the closed gate fold (left) and the open gate fold (right) from curvy fibrils. **c**–**e** Structure and density maps of the closed gate fold. **f**–**h** Structure and density maps of the open gate fold. **c**, **f** Cryo-EM models of closed gate fold (**c**) and open gate fold (**f**), showing the crossover distances. **d**, **g** Cryo-EM density and atomic model of closed-gate fold (**d**) and open gate fold (**g**). The models contain two fragments of transthyretin colored pink (residues Pro 11 to Lys 35) and light sea green (residues Gly 57 or Thr 60 to Asn 124). The mutation site is colored black. The polar channel observed in the closed-gate fold is marked with a dashed rectangle. **e**, **h** Local resolution maps for the closed-gate fold, where blue indicates higher resolution and red indicates lower resolution. **e** The open gate fold (**h**), showing a relatively homogenous local resolution throughout the structure. **i** Close view of the polar channel region, pointing to the hydrophobic interface that is created between the gate of the channel and the residues preceding Ile 84, which could be disturbed in the presence of a serine at position 84. **j** Structural alignment of the closed-gate (in purple) and open gate folds (in orange) highlighting the 180° twist in the residues from Gly 67 backward in the open gate fold. **k** Particle tracing to identify the location of both structure folds within ATTRv-I84S fibrils. Fifteen individual micrographs were used; the representing figure is from one micrograph. Scale bar, 50 nm.

We collected cryo-EM images of cardiac fibrils extracted from the three ATTRv-I84S amyloidosis patients from the same kindred. In each of the three patient samples, two-dimensional (2D) classification discerned classes that differ in their twisting (Fig. 1a). Supplementary Table 3 includes the number of particles corresponding to each class type. The most prevalent species presented a clear twist, here referred to as curvy fibrils. The straight species was not suitable for structure determination by helical reconstruction because it lacked a twist (Fig. 1a and Supplementary Table 3). The three-dimensional (3D) classification of the curvy fibrils in each of the patient samples resulted in at least two structures displaying different local conformations with a total of four distinguishable density maps (Supplementary Fig. 4). Please note that because of resolution, we cannot unambiguously assign the side chain of residue 84 to either isoleucine or serine in any of our models. For simplicity, and with the exception of Fig. 1i, all structure depictions in this paper show the mutation of isoleucine to serine at position 84.

### Polymorphism of fibrils in ATTRv-I84S patient 1

Upon 3D classification of curvy fibrils extracted from ATTRv-I84S patient 1, we obtained five classes that could be visually grouped into two distinct folds (Fig. 1 and Supplementary Fig. 4). We selected two distinct 3D classes for further structural determination (Fig. 1b). The first of the 3D classes was processed and resulted in a density map at a resolution of 3.12 Å (Fig. 1c–e and Supplementary Fig. 5). This population of fibrils has a crossover distance of 689 Å, a twist of –1.26 and, and rise of 4.81 Å (Supplementary Table 3). The built model resembles the structure of the fibrils extracted from the hearts of an ATTRwt patient and an ATTRv-V30M patient, previously determined^21,22^. In this structure, the N- and C-terminal segments encompass residues from Pro 11 to Lys 35 and Gly 57 to Asn 124, respectively (Fig. 1d). We appreciate that each layer of the fibrils contains highly interdigitated interfaces mostly made of hydrophobic residues, also known as steric zippers^26^. There is also a hollow channel that involves residues Leu 58 to Ile/Ser 84 folding into a pentagon-like shape. The interior of this channel is decorated with the side chains of polar residues, and although the structure does not reveal any ordered waters at this resolution, one could speculate that it may be filled with water. In the exterior of this channel, residues from Glu 63 to Ile/Ser 84 are forming close interfaces with other parts of the structure, and residues from Leu 58 to Glu 62 are exposed to the outside of the structure closing the channel, and hence acting as the channel gate (Fig. 1d, i). Based on the structural arrangement of this gate, we classified this structure as the closed gate fold.

The density map from the second 3D class obtained from the curvy fibrils from ATTRv-I84S patient 1 was determined at a resolution of 3.1 Å (Fig. 1f–h and Supplementary Fig. 5). The N- and C-terminal fragments have a residue composition of Pro 11 to Lys 35, and Thr 60 to Asn 124, respectively. These fibrils have a crossover distance of 608 Å, a twist of −1.41°, and rise of 4.8 Å. In contrast to the closed gate fold, the gate in this second 3D class assembles in an extended conformation. This open gate appears twisted by an angle of 180° from Gly 67 backward, resulting in new interactions with the N-terminal fragment (Supplementary Fig. 6). Based on the position of Ile 84 at a hydrophobic interface with Leu 58 and neighbor residues, we anticipate that the mutation to serine could disrupt this interface and then favor the extension and twisting of the gate (Fig. 1i). We classified this new structure as the open gate fold. The structural alignment of the open and closed gate folds emphasizes the differences between the two distinct conformations, with the residues preceding Gly 67 being the most variable (Fig. 1j).

By tracing the fibril segments contributing to these two map densities back to their respective micrographs, we observe that the two polymorphs can coexist within the same fibril (Fig. 1k and Supplementary Fig. 7). A fraction of the filaments contained both structure folds. Because the tracing is only done using well-defined particles that survive the refinement process, the coverage of the filaments is incomplete, thereby, we cannot rule out that the remaining filaments contain one type or both.

### Polymorphism of fibrils in ATTRv-I84S patient 2

**Fig. 2:**
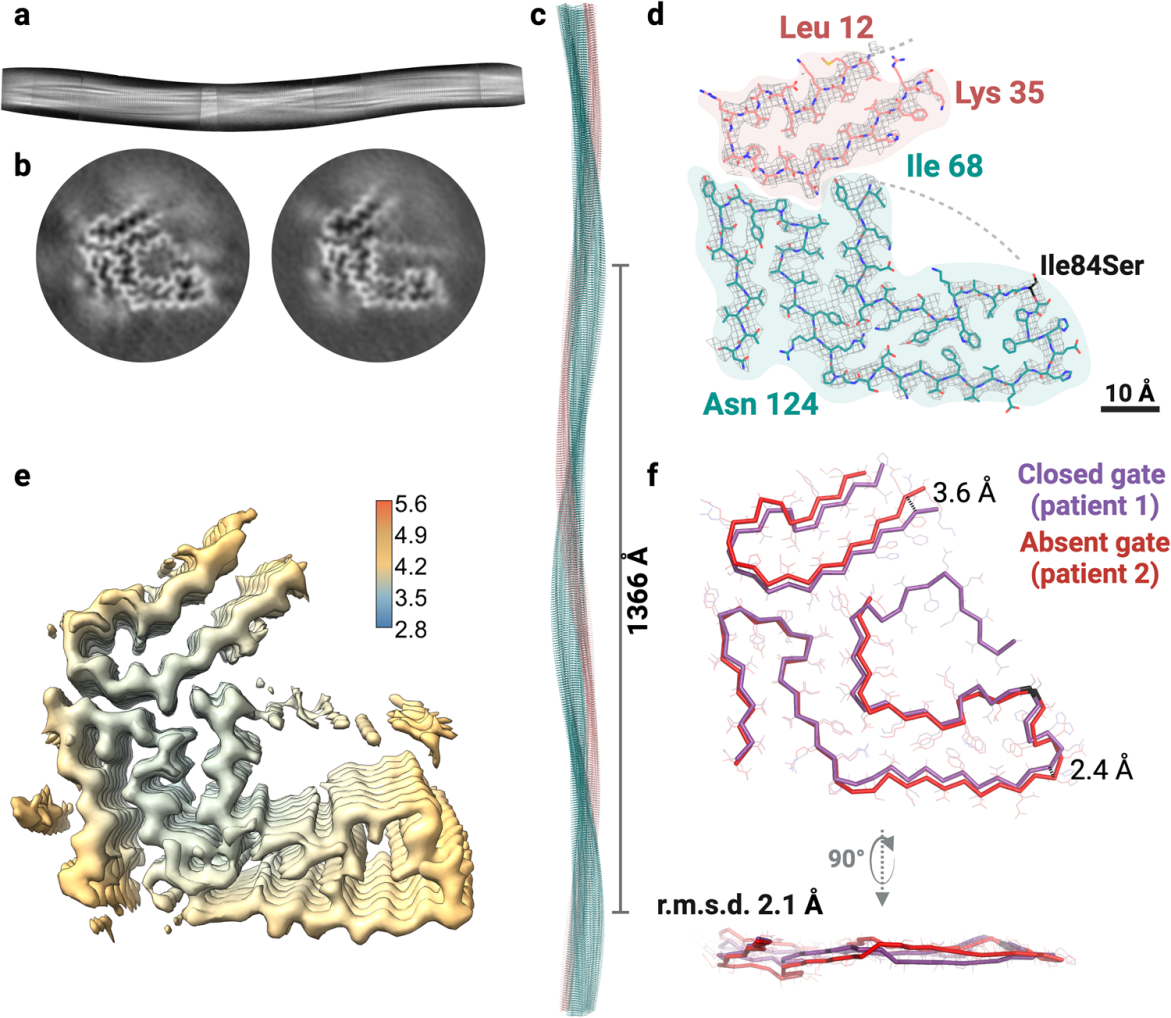
Cryo-EM structure of cardiac fibrils from ATTRv-I84S patient 2. **a** Stitching of the 2D classes of curvy fibrils with the absent gate fold, showing the β-strand separation and the full twist of the fibril. **b** 3D class averages of curvy fibrils showing the closed gate fold (left) and the absent gate fold (right). **c**–**e** Structure and density maps of the absent gate fold. **c** Cryo-EM model of the absent gate fold showing the crossover distance. **d** Cryo-EM density map and atomic model of the absent gate fold. The model contains two fragments of transthyretin colored pink (residues Leu 12 to Lys 35) and light sea green (Ile 68 to Asn 124). The mutation site is colored black. **e** Local resolution map of the absent gate fold, where blue indicates higher resolution and red indicates lower resolution. **f** Structural alignment of the closed gate fold of patient 1 (purple) and the absent gate fold of patient 2 (red) with an overall r.m.s.d. of 2.1 Å. The differences in the tilt of each layer (bottom panel) and the divergence of the two fragments observed in the absent gate fold (with distances of 3.6 Å in the N-terminus and 2.4 Å in the C-terminus) contribute to this r.m.s.d.

We performed 3D classification of curvy ATTRv-I84S fibrils from patient 2, yielding three classes that could be visually grouped into two folds (Fig. 2a, b and Supplementary Fig. 4). One fold resembled the closed gate fold resolved from patient 1; we assumed that their structures are the same and therefore, we did not continue processing this class further for atomic modeling (Fig. 2b and Supplementary Fig. 4). A second 3D class was processed and resulted in a density map with a resolution of 3.8 Å (Fig. 2d, e and Supplementary Fig. 5). This density map had a crossover distance of 683 Å, a twist of −1.3°, and a helical rise of 4.93 Å (Fig. 2c and Supplementary Table 3). This structure comprises of an N-terminal fragment from residue Leu 12 to Lys 35 and a C-terminal fragment extended from Ile 68 to Asn 124. In this fold, the density near the gate was insufficient to model any residues, but appears to block the channel diagonally, resembling the previously determined structure of fibrils obtained from the vitreous humor of an ATTRv-V30M patient (Supplementary Fig. 8)^23^. Because of the lack of density in the polar channel, we classify this structure as the absent gate fold. We compared the closed gate fold from patient 1 to the absent gate fold from patient 2 to highlight the structural differences. We found that the two structures varied mainly in the tilt of each layer and the divergence of the two transthyretin fragments observed in the absent gate fold, leading to an overall r.m.s.d. of 2.1 Å (Fig. 2f).

### Polymorphism of fibrils in ATTRv-I84S patient 3

**Fig. 3:**
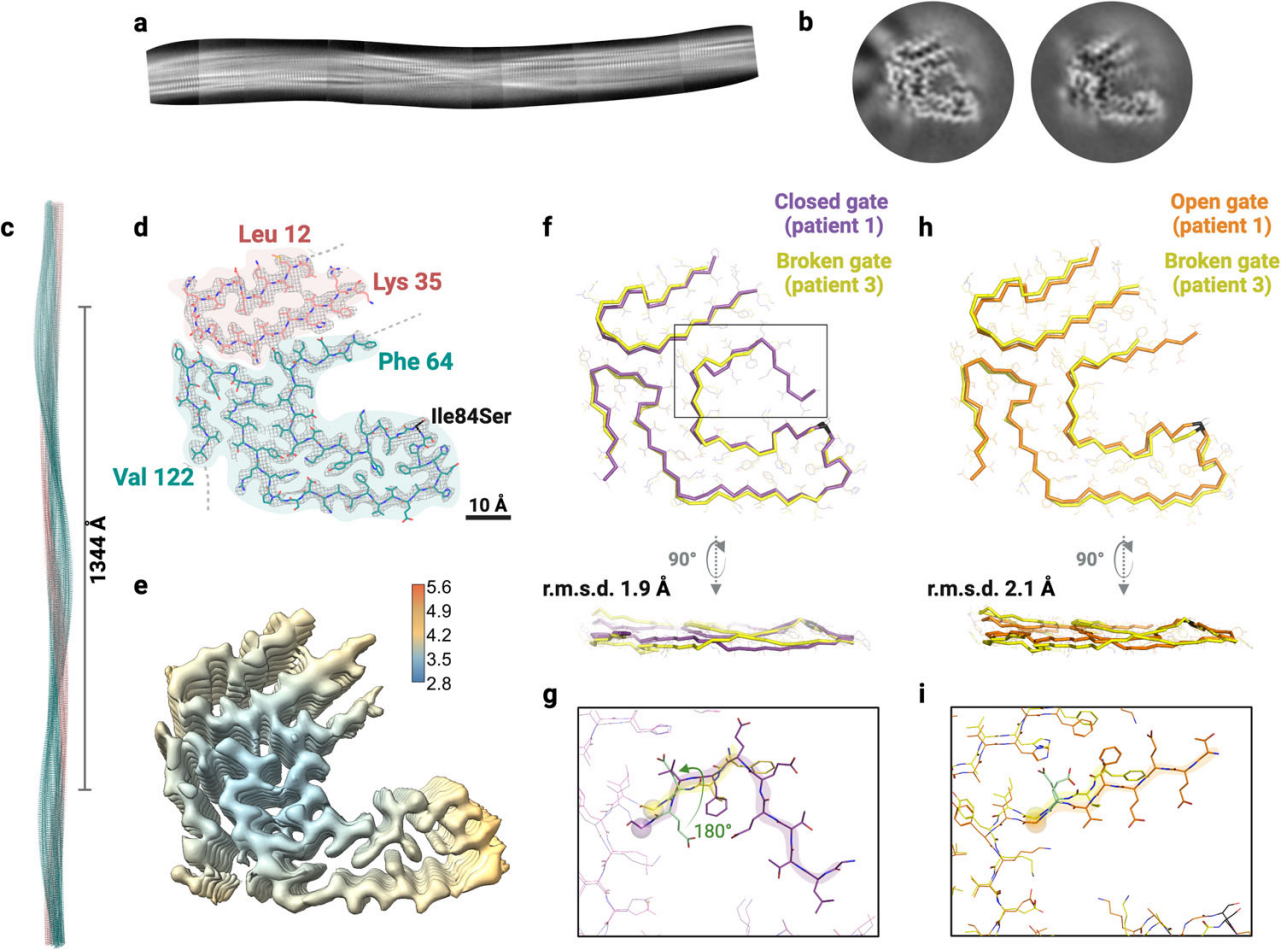
Cryo-EM structure of cardiac fibrils from ATTRv-I84S patient 3. **a** Stitching of the 2D classes of curvy fibrils with the broken gate fold, showing the β-strand separation and the full twist of the fibril. **b** 3D class averages of curvy fibrils with the closed gate fold (left) and the broken gate fold (right). **c**–**e** Density map and model of the broken gate fold. **c** Cryo-EM model showing the crossover distance. **d** Cryo-EM density and atomic models of broken gate fold. The model contains two fragments of transthyretin colored pink (residues Leu 12 to Lys 35) and light sea green (residues Phe 64 to Val 122). The mutation site is colored black. **e** Local resolution map of the broken gate fold, where blue indicates higher resolution and red indicates lower resolution. **f** Structural alignment between the closed gate fold of patient 1 and broken gate fold of patient 3, with an r.m.s.d. of 1.9 Å, highlighting the 180° twist in the gate from Gly 67 backwards (**g**). **h** Structural alignment between the open gate fold of patient 1 (orange) and broken gate fold of patient 3 (yellow), with an r.m.s.d of 2.1 Å, highlighting the residues comprising the gate from Gly 67 backward and their side chains (**i**).

3D classification of fibrils from patient 3 yielded two classes that could be visually grouped into two distinct folds (Fig. 3a, b and Supplementary Fig. 4). Similar to the other two patients, the first class resembled the closed gate fold, and we did not continue processing for atomic modeling (Fig. 3b and Supplementary Fig. 4). Therefore, we utilized the closed gate fold model obtained from patient 1 for comparison purposes. We processed the second class to a resolution of 3.6 Å (Fig. 3 and Supplementary Fig. 5) The fibrils presented with a crossover distance of 672 Å, a twist of −1.3° and a helical rise of 4.96 Å (Fig. 3c and Supplementary Table 3). The N-terminal fragment of the fibril extended from Leu 12 to Lys 35 and the C-terminal fragment extended from residues Phe 64 to Val 122 (Fig. 3d). The density of the C-terminal fragment has an incomplete gate that opens the polar channel to the outside of the structure (Fig. 3d, e). Thus, we classify this structure as the broken gate fold. Like the open gate fold of patient 1, we observe that the gate appears to twist at an angle of 180° from residues Gly 67 backward. This can be observed in the structural alignment between the closed gate fold from patient 1 and the broken gate fold from patient 3, which yielded an overall r.m.s.d. of 1.9 Å. The alignment between the open gate fold from patient 1 and the broken gate fold from patient 3 yielded an overall r.m.s.d of 2.1 Å and highlights the 180° twist on their gates. In these two alignments, we found that the main differences between these structures are in the tilt of the layers across the fibril rather than the fold within each of the layers. For additional structural reference, we have included the maps of all four structures obtained in this study and their local resolutions in Supplementary Fig. 5.

### Structural evaluation of ATTR fibrils

**Fig. 4:**
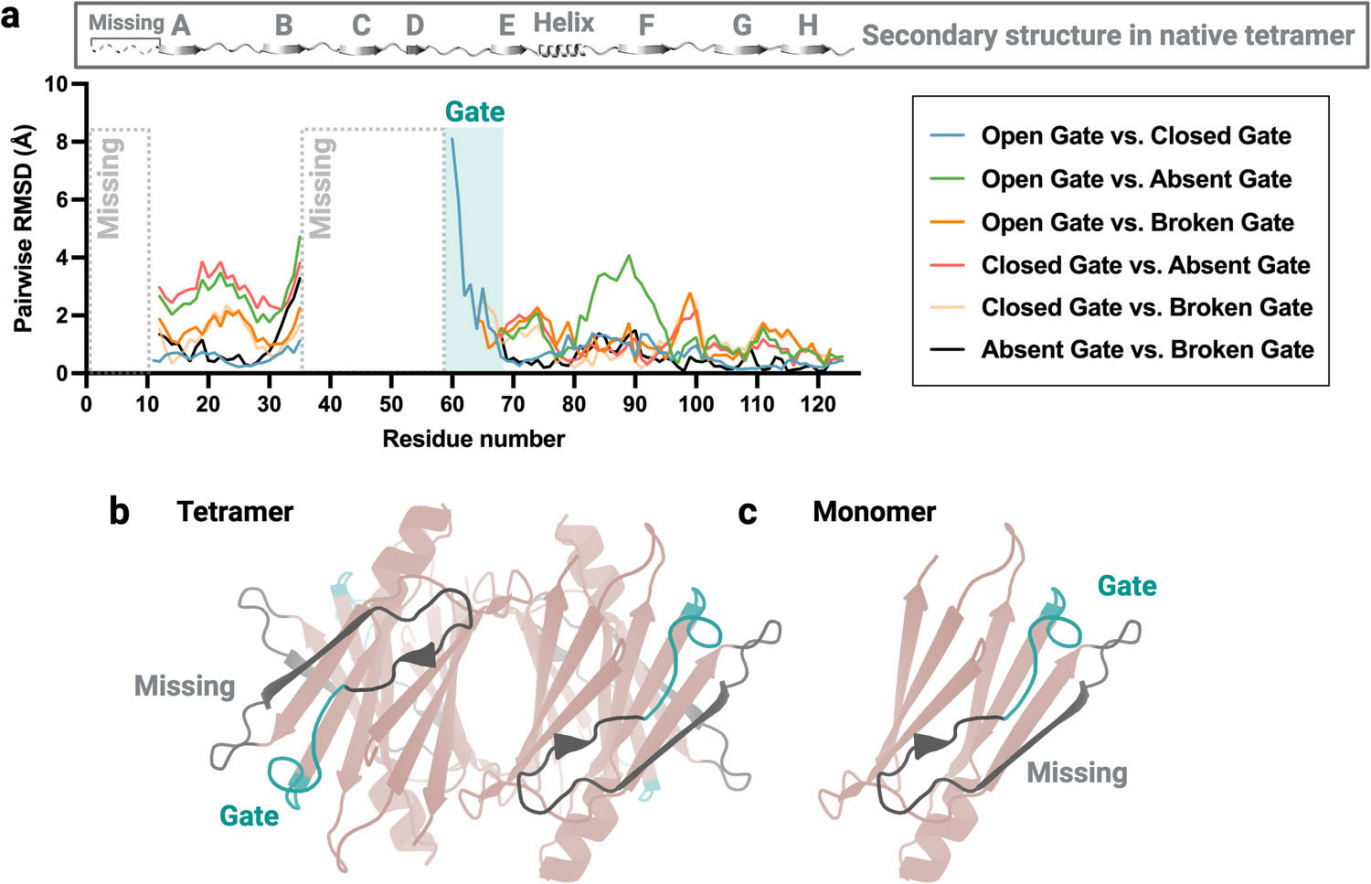
Structural comparison of ATTRv-I84S fibril models. **a** Pairwise r.m.s.d. comparison (C_α_) between the different folds from ATTRv-I84S patients, showing major structural deviations in the gate region and more subtle changes across the protein. The missing region in all fibrils are marked with a dashed rectangle. The residues that contribute to the gate are highlighted in sea green. The top panel depicts the linear secondary structure of the tetrameric transthyretin in its native and functional form. Letters represent distinct β strands. **b**, **c** Location of the missing region and the gate region in the native transthyretin tetramer (**b**) and monomer (**c**) structure. PDB, 4TLT. Residues Gly 1 to Lys 9 are missing in the structures of both tetrameric and amyloid transthyretin.

Although the most noticeable difference between the four ATTRv-I84S fibril folds is found in the gate that opens and closes the polar channel, a closer analysis reveals other structural variations. The pairwise r.m.s.d. analysis of all folds shows that the more drastic structural variations occur in the gate region indeed, with distances that reach almost 8 Å (Fig. 4a). However, there are additional structural differences associated with changes in the tilt of the layers and subtle changes in the folding of each individual layer that result from the divergence of the opposite ends of each layer (Figs. 4a, 1j, 2f, 3f, and 3h).

**Fig. 5:**
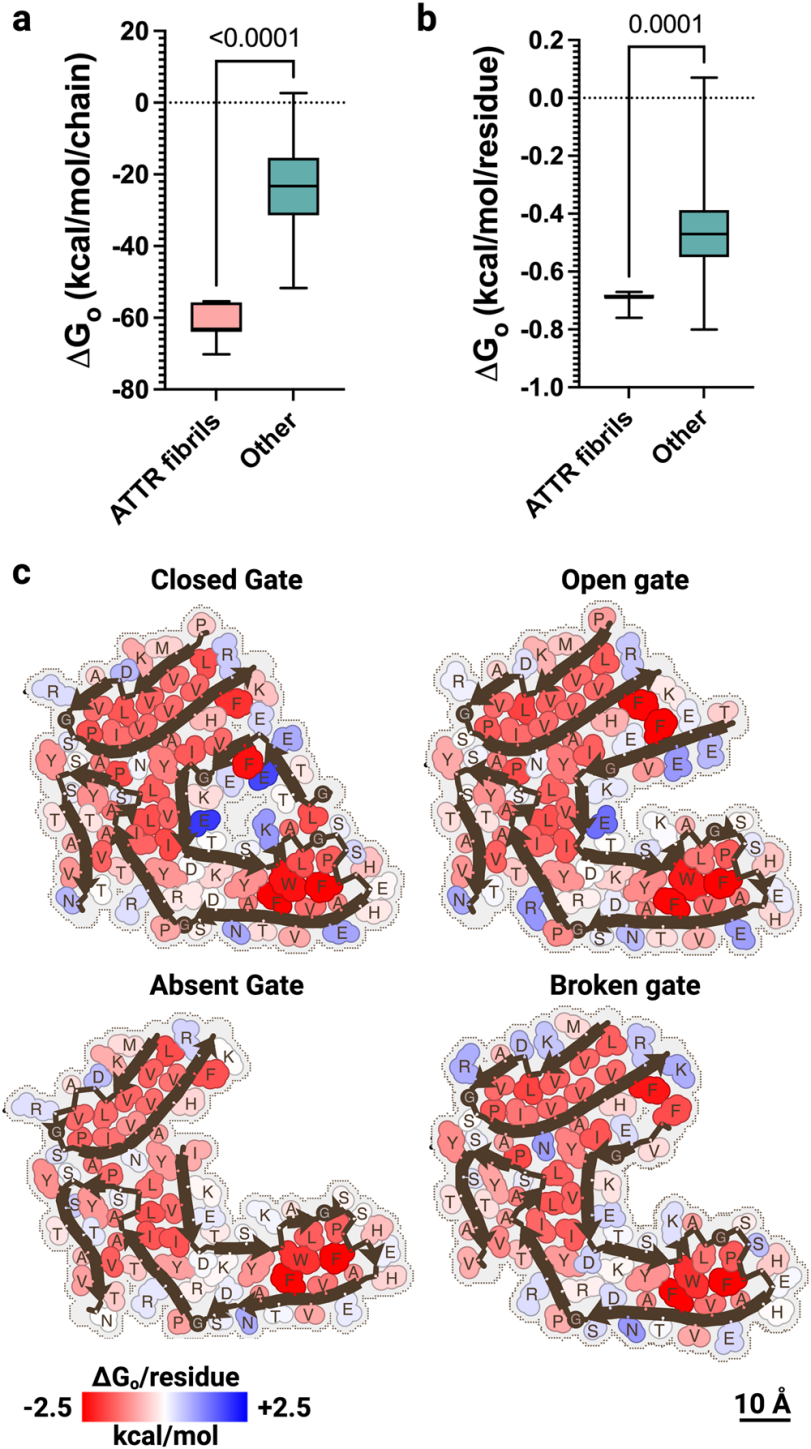
In silico fibril stability in ATTRv-I84S amyloidosis fibrils. **a**, **b** Comparison of average stabilization energies of solvation between ATTR fibrils and all amyloid fibrils reported in the Amyloid Atlas 2023^28^. **a** Stabilization energies by chain. **b** stabilization energies by residue. ATTR fibrils are significantly more stable than the other amyloid structures, considering the energies of both chains and residues. *n* = 7 (ATTR fibrils, ours and from others^21–23^). *n* = 86 (others). Data are presented as Box-and-whisker plots. The horizontal line in each box represents the median value. The 25th–75th percentiles represent the endpoints of the box, with whiskers at minimum and maximum values. Nonparametric two-sided Mann–Whitney *t* test was used for analysis. *P* values are included in the graphs. Graphed data are provided in a Source data file. **c** Representation of stabilization energies per residue of cardiac ATTRv-I84S fibrils determined in this study. Strongly stabilizing side chains are colored red, and destabilizing side chains are colored blue.

Despite the structural differences between the ATTR fibril folds, in silico analysis estimates that they are highly stable relative to other amyloid fibril structures (Fig. 5). The estimated free energy of solvation has been previously used as a proxy to compare structure stabilities^27^. The estimated free energy of solvation of these fibrils per chain ranges from –55.4 and –70.2 kcal/mol. Using this property, we compared their stability to all the amyloid structures reported in the Amyloid Atlas by January 2023 and found that ATTR fibrils on average are estimated to be significantly more stable than other fibrils, both by residue and chain (Fig. 5a, b)^28^. Analysis by residue reveals that the stability of ATTR fibrils results from the contribution of three pockets—the inner interface of the hairpin formed between Val 14 and Val 32, the inner pocket of the arch formed between residues Trp 79 and Phe 95, and the triquetra that connects the C- and N-terminal fragments together (Fig. 5c). Most ionizable residues of ATTR fibrils are exposed to the outside, thereby enabling neutralization by other interactions that also contribute to the fibril stability (Supplementary Fig. 6). These include hydrogen bonding between residues from different fragments, π–π stacking of aromatic residues, hydrogen bonding from the stacking of asparagine residues along the fibril, and hydrogen bonding of exposed ionizable residues within the same chain (Supplementary Fig. 6a–d). Albeit the structural changes found in the closed gate and open gate folds, these do not result in changes in the estimated free energy of solvation, thereby suggesting that these variations in the polar channel may not affect stability drastically.

The residue composition of all the ATTRv-I84S structures is overall similar. While the composition of the N-terminal fragment is similar in all four structures, we observe large variations at the start of the C-terminal fragment around the region of the polar channel (Supplementary Fig. 9). Overall, all hydrophobic residues face the inside of the structures and contribute to the formation of steric zippers, and polar and charged residues face the outside of the structures and/or contribute to the formation of salt bridges and hydrogen bonds (Supplementary Figs. 6 and 9). These interactions likely contribute to the high stability discussed above.

The comparison of the secondary structure of ATTRv-I84S fibrils to native transthyretin reveals significant changes that would entail major unfolding and reassembly of the tetramer (Fig. 4 and Supplementary Fig. 10). While all four ATTRv-I84S fibril folds share similar secondary structures with minor variations, they do not coincide with the secondary structure profile of the native tetrameric transthyretin (Supplementary Fig. 10). Residues Ala 36 to His 56, missing in the structure of ATTR fibrils, are located on the outside of the tetrameric protein and contained both structured (β-strands) and unstructured (loop) regions surrounding strands C and D (Supplementary Fig. 10 and Fig. 4b, c). The residues involving the gate (Leu 58 to Gly 67) of the ATTR fibrils are also located on the outside of the tetrameric protein and are unstructured (Supplementary Fig. 10 and Fig. 4b, c). The significance of this is yet to be determined.

Despite these structural differences, all ATTRv-I84S fibril structures share a common core that includes a fragment from the N-terminus (from residues Leu 12 to Lys 35) and the C-terminus (from residues Gly 67 to Val 122). Tryptic ESI/MS mass spectrometry analysis show that the fibril composition and proteolytic sites are comparable (Supplementary Table 2). LC/MS QTOF mass spectrometry confirms the presence of the fragment Leu 58 to Glu 127 in all samples (Supplementary Table 1 and Supplementary Fig. 3). These results suggest that the structural differences may be attributed to the presence of the mutation rather than differential proteolytic events.

## Discussion

**Fig. 6:**
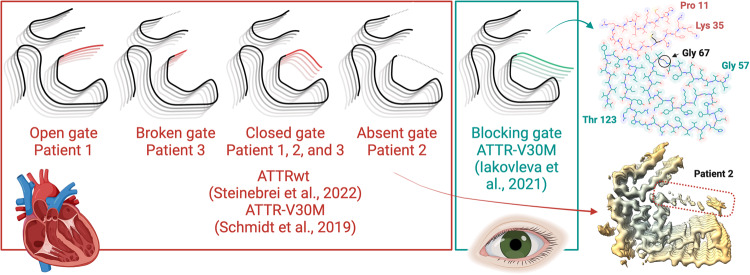
A schematic representation of the fibril polymorphism observed in ATTR fibrils. Blocking gate structure obtained from PDB 7OB4. Schemes generated on BioRender.

Multiple structural studies of amyloid fibrils extracted from patients suffering from amyloid neurodegenerative diseases such as tauopathies and synucleinopathies suggest an association between each disease and the amyloid fibril structure^15,17,29,30^. This association has not yet been studied in systemic amyloid conditions such as ATTR amyloidosis. Here we determine the fibril structures from three ATTR amyloidosis patients carrying the mutation I84S (*pATTRv-I104S) from the same kindred^24^. Our study reveals that despite the consistent clinical presentation associated with ATTRv-I84S patients, historically classified as familial amyloidotic polyneuropathy type II^24,31^, their cardiac fibrils exhibit structural heterogeneity amongst different individuals (Fig. 6). We found that ATTRv-I84S fibrils share a common core that includes a fragment from the N-terminus (from residues Leu 12 to Lys 35) and the C-terminus (from residues Gly 67 to Val 122). However, ATTRv-I84S fibrils display major differences in the C-terminal fragment that acts as a gate of the polar pocket; this pocket becomes a channel when the gate closes it (Fig. 1). The four structures determined in this study vary in the assembly of this gate, and we classify them as the closed gate, the open gate, the absent gate, and the broken gate folds (Fig. 6). Notably, we found that all patients had at least two types of fibril folds, including the closed gate fold and a second fold that is specific to the patient (Figs. 1–3), and both can coexist within the same fibril (Fig. 1k and Supplementary Fig. 7). The implications of these discoveries are discussed below.

Amyloid polymorphism has potential implications in systemic amyloidosis. In this study, we focus on the structure of cardiac ATTR fibrils to evaluate structural polymorphism in the heart of heterozygous ATTRv-I84S patients. Patients with the ATTRv-I84S mutation follow a distinct but almost consistent pattern of disease progression: carpal tunnel syndrome typically occurring in the early 30–40 s, followed by a generalized peripheral polyneuropathy, vitreous humor opacities, and ultimately cardiac manifestations leading to death due to cardiac arrest in the 50–60s^24,32^. Our study demonstrates that despite the consistent phenotype associated with ATTRv-I84S mutation, we observe local polymorphism within and between patients from the same kindred (Fig. 6). In contrast, the structures of fibrils from patients suffering from neurodegenerative amyloid diseases, also referred to as brain amyloidosis, are disease-specific. In other words, all individuals with Alzheimer’s disease, for instance, present the same tau fibril fold^12^, suggesting that every amyloid disease is associated with specific fibril polymorph(s)^17^. These findings may be attributed to the fact that most of these structures contain only wild-type tau and α-synuclein^15,17^. A recent study reveals that mutant α-synuclein fibrils from one individual suffering from early-onset Parkinsonism (known as Juvenile Onset Synucleinopathy), depict a distinct fold that differs from the folds of wild-type α-synuclein^29^. In our study, although the ATTR fibrils were obtained from the same organ, the heart, from patients with the same disease-associated mutation, ATTRv-I84S, they adopt different structures with local variations that are individual-specific.

It may be possible that this inconsistency may be attributed to the systemic nature of ATTR amyloidosis. Fibril polymorphism in systemic diseases has been previously investigated in an independent study using light chain fibrils from an AL amyloidosis patient^33^. Similar to what we observe, different AL fibril structures can coexist within the same fibril in the same individual. On the other hand, serum amyloid A (SAA) fibrils from two AA amyloidosis patients show distinct structures when obtained from vascular and glomerular regions of the kidney, and the involvement of the two regions is associated with distinct clinical phenotypes^34,35^. Overall, the structural landscape of fibrils in systemic amyloidosis may be diverse and requires further studies on each individual disease. Based on our observations, we speculate that fibril polymorphism in ATTR amyloidosis, when it occurs within the same disease and the same organ, may be individual-specific and driven by the presence of the mutation.

Polymorphism has additional implication in Type A ATTR amyloidosis. Several studies suggest a potential connection of ATTR amyloid fibril histopathology with clinical presentation. Histopathological studies of ATTR fibrils reveal two distinct fibril types: type A, composed of full-length and fragmented transthyretin, and type B, composed exclusively of full-length transthyretin^25^. Type A and B fibrils are associated with differential clinical presentations in ATTRv-V30M amyloidosis patients, and with a more variable clinical outcome in type A patients^36^. In previous studies, we showed that the presence of fragmented transthyretin in ATTR fibrils has a positive correlation with their potential to seed fibril formation^37^. Consistently, type A ATTR patients develop rapid cardiac deposition of wild-type transthyretin after liver transplantation^38^. To date, all the samples used for cryo-EM structure reconstruction of ATTR fibrils in this paper (Fig. 6) and from others^21–23^ contain type A fibrils. Mass spectrometry of the ATTRv-I84S fibrils in our study show that all three patient samples have similar fragment composition (Supplementary Tables 1 and 2 and Supplementary Fig. 3), including near the polar pocket region. This finding and the strategic location of the I84S mutation (Fig. 1i) suggest that rather than differential proteolytic events, the I84S mutation drives the observed local structural polymorphism. Thus, the structural differences in type A fibrils may be dictated by mutations or other unknown factors. The association between this structural heterogeneity and the clinical variability observed in type A patients remains to be studied.

The implications of the polar pocket and its structural variability represent a puzzle in the pathology of ATTR amyloidosis. All ATTR fibril structures from this study as well as previously published structures of ATTRwt and ATTRv-V30M share the presence of a polar pocket that is lined with ionic residues from the C-terminal fragment of transthyretin^21–23^. In the closed gate fold from patient 1 in this work and in previously published ATTRv-V30M and ATTRwt fibrils, this segment surrounds and closes the polar channel in a pentagon-like shape that encompasses residues Leu 58 to Ile 84 (Figs. 1d and  6)^21,22^. In the open gate fold of ATTRv-I84S fibrils from patient 1, the gate is extended (Figs. 1g and  6). In the absent gate fold of patient 2, this segment is absent, thus exposing the polar residues to the surrounding solvent (Figs. 2d and  6). However, we do observe a low density that appears to cross the channel diagonally (Fig. 2e), similar to what is observed in the structure of ATTRv-V30M fibrils from the vitreous humor (Supplementary Fig. 8)^23^. In patient 3, the gate appears broken and begins from residue Phe 64 (Figs. 3d and  6). In all these cases, the pivotal residue Gly 67 governs the conformational polymorphism of the polar pocket of ATTR fibrils, acting as a hinge that opens or closes it into a channel^28^. We note that this conformational variability in the polar channel, and the resulting changes in the nature of the interactions of the gate (Supplementary Fig. 6) does not affect ATTR fibril stability drastically (Fig. 5)^21–23^. Our in silico study shows that all ATTR fibrils are estimated to be, on average, the most stable amyloid assemblies when compared to other amyloid fibrils (Fig. 5a). The estimated energy of solvation shows that in all fibril polymorphs from our study, the core contributes predominantly to the stability of the fibrils with the channel providing only a minor contribution, perhaps explaining why the channel can be found in different conformations. In addition, our mass spectrometry studies suggest that the extension or closing of the gate does not affect fibril proteolysis, previously associated with Type A fibrils^39^. These observations question the role of the polar pocket in amyloidogenesis and/or pathogenesis in ATTR amyloidosis. Perhaps, the content of the channel or pocket could play a role in pathology as speculated in the case of neurodegenerative diseases^13^. Many other fibril structures also contain polar or hydrophobic channels with unknown functions^28^. Future studies may shed further light on the role of the polymorphism of the polar pocket and/or channel and its association with ATTR disease pathology, if there is any.

Our study contributes to the understanding of protein aggregation in ATTR amyloidosis. Our mass spectrometry analysis and structures suggest that, similar to previously described ATTR structures, ATTRv-I84S fibrils are compatible with a proposed proteolytic mechanism in where proteolysis occurs after fibril formation^21,22^. The presence of two individual N- and C-fragments encompassing the core of the ATTR fibril structures at a 1:1 ratio makes it unlikely for proteolytic event to happen before fibril formation.

The ATTRv-I84S fibrils structures also raise interesting questions about the role of the I84S mutation in ATTR amyloid formation and pathogenesis. A previous study shows that I84S mutation lowers the disassociation barrier of the tetramers into monomers^40^. Our study shows that in addition to this role, the I84S mutation may also drive fibril polymorphism. Because of its strategic location, the residue Ile 84 contributes to a hydrophobic interface (Fig. 1i) that closes the polar channel. This interface could be disrupted by the presence of a more polar residue, serine, thereby triggering the formation of variable gate conformations. We also observe that all three heterozygous ATTRv-I84S patients present with fibrils adopting a closed gate fold, in addition to their unique folds. We speculate that the prevalence of this population of closed gate fibrils may be influenced by the content of wild-type transthyretin. Nevertheless, a common core structure that includes a fragment from the N-terminus (from residues Leu 12 to Lys 35) and the C-terminus (from residues Gly 67 to Val 122) in all fibril folds may serve an important role for amyloid formation. The co-existence of different local conformations within the same fibril also suggests that the common core in the structure may be sufficient to promote elongation and seeding of the different structures. Overall, our results suggest that the mechanism of aggregation of transthyretin into different structures with local variations may be determined by the dissociation of tetramers as well as mutation-induced structural alterations.

Our study has limitations. Because of our limited access to the clinical histories’ of these patients, correlations between disease progression and structure cannot be discerned. In addition, in this present study, we have looked at heart samples from three patients with one mutation. Future studies that involve structural analysis of more mutations and other organs, as well as experimental validation of their stability, can shed further light on the role of mutations and influence of the tissue microenvironment in structural polymorphism in ATTR amyloidosis.

In summary, our cryo-EM study demonstrates the existence of local structural variations in ATTRv-I84S fibrils. Our study provides further evidence that one disease may associate with multiple fibril structures in systemic amyloidoses. We also discuss a possible mechanism through which mutations affect ATTR fibril polymorphism, by driving structural changes in the polar pocket, which do not reduce their extraordinary stability. Overall, our study opens questions about the structural implications of mutations in ATTR amyloidosis and other amyloid conditions.

## Methods

### Patients and tissue material

We obtained cardiac tissues from three ATTR patients carrying I84S mutation, one patient carrying wild-type ATTR, and one patient carrying the V30M mutation (*n* = 5). All samples were postmortem. ATTRv-I84S patients 1 and 3 were male and patient 2 was a female. The patient with wild-type ATTR was 78 years male with transplanted heart. The patient with type B ATTR was male caring V30M TTR mutation with peripheral and autonomic neuropathy and died at the age of 42 years. All patients were in their 50’s at the time of collection. Specimens from the left ventricle were obtained from the laboratory of Dr. Merrill D. Benson at the University of Indiana. The Office of the Human Research Protection Program granted exemption from Internal Review Board review because all specimens were anonymized.

### Extraction of amyloid fibrils from human cardiac tissue

Ex-vivo preparations of amyloid fibrils were obtained from fresh-frozen human tissue or lyophilized fibrillar extract as described earlier^21^. Briefly, ∼200 mg of frozen cardiac tissue per patient was thawed at room temperature and cut into small pieces with a scalpel. The minced tissue or ∼100 mg of lyophilized fibrillar extract was suspended into 1 mL Tris-calcium buffer (20 mM Tris, 150 mM NaCl, 2 mM CaCl_2_, 0.1% NaN_3_, pH 8.0) and centrifuged for 5 min at 3100×*g* and 4 °C. The pellet was washed in Tris-calcium buffer four additional times. After the washing, the pellet was resuspended in 1 mL of 5 mg/mL collagenase solution (collagenase was dissolved in Tris-calcium buffer) and incubated overnight at 37 °C, shaking at 400 rpm. The resuspension was centrifuged for 30 min at 3100×*g* and 4 °C and the pellet was resuspended in 1 mL Tris–ethylenediaminetetraacetic acid (EDTA) buffer (20 mM Tris, 140 mM NaCl, 10 mM EDTA, 0.1% NaN_3_, pH 8.0). The suspension was centrifuged for 5 min at 3100×*g* and 4 °C, and the washing step with Tris–EDTA was repeated nine additional times. All the supernatants were collected for further analysis, when needed. After the washing, the pellet was resuspended in 200 μL ice-cold water supplemented with 5-10 mM EDTA and centrifuged for 5 min at 3100×*g* and 4 °C. This step released the amyloid fibrils from the pellet, which were collected in the supernatant. EDTA helped solubilize the fibrils. This extraction step was repeated 3–5 additional times. The material from the various patients was handled and analyzed separately.

### Negative-stained transmission electron microscopy

Amyloid fibril extraction was confirmed by transmission electron microscopy as described^41^. Briefly, a 3- μL sample was spotted onto a glow-discharged carbon film 300-mesh copper grid (Electron Microscopy Sciences), incubated for 2 min, and gently blotted onto a filter paper to remove the solution. The grid was negatively stained with 5 µL of 2% uranyl acetate for 2 min and gently blotted to remove the solution. Another 5 μL uranyl acetate was applied onto the grid and immediately removed. An FEI Tecnai 12 electron microscope at an accelerating voltage of 120 kV was used to examine the specimens.

### Recombinant protein expression and purification

Recombinant protein samples were prepared as described previously^41^. Briefly, monomeric transthyretin (MTTR) was expressed in *Escherichia coli* Rosetta™ (DE3) pLysS Competent Cells (MilliporeSigma) and purified by affinity in a HisTrap column (GE Healthcare Life Science). Peak fractions were combined and further purified by size exclusion chromatography on a Superdex S75 prep grade column (GE Healthcare Life Science) in sodium phosphate–EDTA buffer (10 mM sodium phosphate pH 7.5, 100 mM KCl, and 1 mM EDTA). Peak fractions were pooled and stored at −80 °C.

### Amyloid seeding assays

Amyloid fibril extracts were used to seed the formation of new fibrils from recombinant MTTR as we described previously^37^. Briefly, we further purified the extracts by treatment with 1% sodium dodecyl sulfate (SDS) in sodium phosphate–EDTA buffer and centrifugation at 15,000×*g* for 20 min. This purification process was repeated two times, and soluble fractions were discarded. The sample was washed with sodium phosphate–EDTA (without the addition of 1% SDS) buffer three times by centrifugation and sonicated using bath sonicator in cycles of 5 s on and 5 sec off for a total of 10 min with minimum amplitude 30 (Q700 sonicator, Qsonica). The total protein content in the seed preparation was measured by the Micro BCA™ Protein Assay Kit (Thermo Fisher Scientific) 2% (w/w) seeds were added to 0.5 mg/mL recombinant MTTR in a final volume of 200 μL containing 10 μM thioflavin T (ThT) and 1× PBS (pH 7.4). ThT fluorescence emission was measured at 482 nm with absorption at 440 nm in a FLUOstar Omega (BMG LabTech) microplate reader. Plates (384 Well Optical Btw Plt Polybase Black w/o Lid Non-Treated PS, Thermo Fisher Scientific) were incubated at 37 °C with cycles of 9 min shaking (700 rpm double orbital) and 1 min rest throughout the incubation. Measurements were taken every 10 min (bottom read) with a manual gain of 1000-fold. Fibril formation was confirmed by transmission electron microscopy as described above.

### Western blotting of extracted ATTR fibrils

Western blotting was performed on the extracted fibrils to confirm fibril type^38^. Briefly, 0.5 µg of fibrils were dissolved in a tricine SDS sample buffer, boiled for 2 min at 85 °C, and run on a Novex™ 16% tris-tricine gel system using a Tricine SDS running buffer. TTR type was determined by transferring the gel contents to a 0.2-µm nitrocellulose membrane and probing with a primary antibody (1:1000) directed against the C-terminal region of the wild-type TTR sequence (GenScript). Horseradish peroxidase-conjugated goat anti-rabbit IgG (Invitrogen, at 1:1000) was used as a secondary antibody. Promega Chemiluminescent Substrate (Promega) was used according to the manufacturer’s instructions to visualize TTR content.

### Cryo-EM sample preparation, data collection, and processing

Freshly extracted fibril samples were applied to glow-discharged Quantifoil R 1.2/1.3, 300-mesh, Cu grids, blotted with filter paper to remove excess sample, and plunged frozen into liquid ethane using a Vitrobot Mark IV (FEI). Cryo-EM samples were screened on either the Talos Arctica or Glacios at the Cryo-Electron Microscopy Facility (CEMF) at University of Texas Southwestern Medical Center (UTSW), and the final datasets were collected on a 300 kV Titan Krios microscope (FEI) at two different facilities: the CEMF and the Stanford-SLAC Cryo-EM Center (S^2^C^2^) (Supplementary Table 3). Pixel size, frame rate, dose rate, final dose, and number of micrographs per sample are detailed in Supplementary Table 3. Automated data collection was performed by SerialEM software package^42^.

The raw movie frames were gain-corrected, aligned, motion-corrected and dose-weighted using RELION’s own implemented motion correction program^43^. Contrast transfer function (CTF) estimation was performed using CTFFIND 4.1^44^. All steps of helical reconstruction, three-dimensional (3D) refinement, and post-process were carried out using RELION 4.0^45^.

All filaments were manually picked using EMAN2 e2helixboxer.py^46^. Particles were first extracted using a box size of 1024 with an inter-box distance of 3 asymmetrical units at 4.75 Å apart, and 256 pixels with an inter-box distance of 1 asymmetrical unit at 4.75 Å apart. 2D classification of 1024-pixel particles was used to estimate the helical parameters. 2D classifications of 256-pixel particles were used to select suitable particles for further processing. Fibril helix is assumed to be left-handed. We performed 3D classifications with an average of ∼30k to 40k particles per class to separate filament types using an elongated Gaussian blob as an initial reference. Particles potentially leading to the best-reconstructed map were chosen for 3D auto-refinements. CTF refinements and Bayesian polishing were performed to obtain higher resolution. Final maps were post-processed using the recommended standard procedures in RELION. The final subset of selected particles was used for high-resolution gold-standard refinement as described previously^47^. The final overall resolution estimate was evaluated based on the FSC at 0.143 threshold between two independently refined half-maps (Supplementary Fig. 11)^48^.

### Model building

The refined map of patient 1 was further sharpened using phenix.auto_sharpen at the resolution cutoff^49^. A previously published model of ATTRv-T60A (pdb code 8E7G) was used as the template to build all near-atomic resolution models. Mutations, rigid body fit zone, and real space refine zone were performed to obtain the resulting models using COOT^50^. All the statistics are summarized in supplementary Table 3.

### Filament tracing

The coordinates of fibril segments (rlnCoordinateX and rlnCoordinateY) of the final Refine3D were extracted from the relion file run_data.star. These coordinates were plotted onto XY-graphs, and the graphs were adjusted to match the corresponding micrographs dimensions. It is important to note that only the well-defined segments of fibrils were included for reconstruction, omitting any subpar segments. As a result, the positions of these well-defined segments, especially when they belong to the same fibril, may appear interspersed as illustrated in Fig. 1k and Supplementary Fig. 7.

### Mass spectrometry (MS) sample preparation, data acquisition and analysis

For tryptic MS analysis, 0.5 µg of extracted ATTR fibrils (three ATTRv-I84S and one wild-type control) were dissolved in a tricine SDS sample buffer, boiled for 2 min at 85 °C, and run on a Novex™ 16% tris-tricine gel system using a Tricine SDS running buffer. Gel was stained with Coomassie dye, destained and ATTR smear was cut from the gel. The sample was sent for MS analysis. Samples were digested overnight with trypsin (Pierce) following reduction and alkylation with DTT and iodoacetamide (Sigma-Aldrich). The samples then underwent solid-phase extraction cleanup with an Oasis HLB plate (Waters), and the resulting samples were injected onto an Q Exactive HF mass spectrometer coupled to an Ultimate 3000 RSLC-Nano liquid chromatography system. Samples were injected onto a 75 µm i.d., 15-cm long EasySpray column (Thermo) and eluted with a gradient from 0-28% buffer B over 90 min. Buffer A contained 2% (v/v) ACN and 0.1% formic acid in water, and buffer B contained 80% (v/v) ACN, 10% (v/v) trifluoroethanol, and 0.1% formic acid in water. The mass spectrometer operated in positive ion mode with a source voltage of 2.5 kV and an ion transfer tube temperature of 300 °C. MS scans were acquired at 120,000 resolution in the Orbitrap and up to 20 MS/MS spectra were obtained in the ion trap for each full spectrum acquired using higher-energy collisional dissociation (HCD) for ions with charges 2–8. Dynamic exclusion was set for 20 s after an ion was selected for fragmentation.

Raw MS data files were analyzed using Proteome Discoverer v3.0 SP1 (Thermo), with peptide identification performed using a semitryptic search with Sequest HT against the human-reviewed protein database from UniProt. Fragment and precursor tolerances of 10 ppm and 0.02 Da were specified, and three missed cleavages were allowed. Carbamidomethylation of Cys was set as a fixed modification, with oxidation of Met set as a variable modification. The false-discovery rate (FDR) cutoff was 1% for all peptides.

For Intact protein mass analysis, 5 µg of extracted ATTR were disaggregated with 8 M Guanidinium Hydrochloride. Samples were desalted and analyzed by LC/MS, using a Sciex X500B QTOF mass spectrometer coupled to an Agilent 1290 Infinity II HPLC. Samples were injected onto a POROS R1 reverse-phase column (2.1 × 30 mm, 20 µm particle size, 4000 Å pore size) and desalted. The mobile phase flow rate was 300 µL/min, and the gradient was as follows: 0-3 min: 0% B, 3–4 min: 0–15% B, 4–16 min: 15–55% B, 16–16.1 min: 55–80% B, 16.1–18 min: 80% B. The column was then re-equilibrated at initial conditions prior to the subsequent injection. Buffer A contained 0.1% formic acid in water, and buffer B contained 0.1% formic acid in acetonitrile.

The mass spectrometer was controlled by Sciex OS v.3.0 using the following settings: Ion source gas 1 30 psi, ion source gas 2 30 psi, curtain gas 35, CAD gas 7, temperature 300 °C, spray voltage 5500 V, declustering potential 135 V, collision energy 10 V. Data was acquired from 400 to 2000 Da with a 0.5 s accumulation time and four time bins summed. The acquired mass spectra for the proteins of interest were deconvoluted using Bio Tool Kit within Sciex OS in order to obtain the molecular weights. Peaks were deconvoluted over the entire mass range of the mass spectra, with an output mass range of 7000–9000 Da, using low input spectrum isotope resolution.

The mass spectrometry proteomics data have been deposited to MassIVE (a member of ProteomeXchange)^51^.

### Stabilization energy calculation

The stabilization energy per residue was calculated by the sum of the products of the area buried for each atom and the corresponding atomic solvation parameters (Fig. 5)^52,53^. The overall energy was calculated by the sum of energies of all residues, and assorted colors were assigned to each residue, instead of each atom, in the solvation energy map.

### Statistical analysis

Statistical analysis of fibril stability was performed with Prism 9 for Mac (GraphPad Software) using an unpaired *t* test. All samples were included in the analysis, and all measurements are displayed in the graphs.

### Reporting summary

Further information on research design is available in the Nature Portfolio Reporting Summary linked to this article.

### Supplementary information

Supplementary Information

Peer Review File

Reporting Summary

### Source data

Source data

## Data Availability

Structural data have been deposited into the Worldwide Protein Data Bank (wwPDB) and the Electron Microscopy Data Bank (EMDB) with the following EMD accession codes: EMD-41171 (Closed Gate, Patient 1), EMD-41172 (Open Gate), EMD-26685 (Absent Gate), EMD-27323 (Broken Gate), and PDB accession codes: 8TDN (Closed Gate, Patient 1), 8TDO (Open Gate), 8E7E (Absent Gate), 8E7J (Broken Gate). The PDB accession codes for the previously reported coordinates of ATTRv-V30M fibrils from vitreous humor and heart are 7OB4 and 6SDZ, respectively. All data generated or analyzed during this study that support the findings are available within this published article and its supplementary data files. MS Data are available via MassIVE (a member of ProteomeXchange) with identifier MSV000093061 [https://massive.ucsd.edu/ProteoSAFe/dataset.jsp?task=019f119d13b747d09e22bc352e41b7d6] for QTOF data and MSV000093062 [https://massive.ucsd.edu/ProteoSAFe/dataset.jsp?task=3f0caffc5cb74a1f85e5b9e6ce8a8b5a] for LC-MS/MS data. Graphed data is provided in the source data file. Cardiac specimens were obtained from the laboratory of late Dr. Merrill D. Benson at Indiana University. These specimens are under a material transfer agreement with Indiana University and cannot be distributed freely. Source data are provided with this paper.
