# Supplementary material for: Structural polymorphism of amyloid fibrils in ATTR amyloidosis revealed by cryo-electron microscopy
Source: Nat Commun. 2024 Jan 17;15:581. doi: 10.1038/s41467-024-44820-3 (PMC10794703; doi:10.1038/s41467-024-44820-3)
Supplement: Supplementary file 1 — Supplementary Information [file 41467_2024_44820_MOESM1_ESM.pdf]

Title: Structural polymorphism of amyloid fibrils in ATTR amyloidosis revealed by cryo-electron microscopy

Authors: Binh An Nguyen[1,2,3$], Virender Singh[1,2,3$], Shumaila Afrin[1,2,3$], Anna Yakubovska[1,2,3], Lanie Wang[1,2,3], Yasmin Ahmed[1,2,3], Rose Pedretti[1,2,3], Maria del Carmen Fernandez-Ramirez[1,2,3], Preeti Singh[1,2,3], Maja Pękała[1,2,3], Luis O. Cabrera Hernandez[1,2,3], Siddharth Kumar[1,2,3], Andrew Lemoff[4], Roman Gonzalez-Prieto[5], Michael Sawaya[6], David Eisenberg[6], Merrill Douglas Benson[7], Lorena Saelices[1,2,3*]

[$]These authors contributed equally.

Affiliations:

[1]*Center for Alzheimer's and Neurodegenerative Diseases, University of Texas Southwestern Medical Center (UTSW), Dallas, TX, USA.*

[2] *Department of Biophysics, University of Texas Southwestern Medical Center (UTSW), Dallas, TX, USA.*

[3] *Peter O'Donnell Jr Brain Institute, University of Texas Southwestern Medical Center (UTSW), Dallas, TX, USA.*

[4]*Department of Biochemistry, University of Texas Southwestern Medical Center, Dallas, TX, USA*

[5]*Andalusian Center for Molecular Biology and regenerative Medicine (CABIMER), Universidad de Sevilla-CSIC-Universidad-Pablo de Olavide, Departmento de Biología Celular, Facultad de Biología, Universidad de Sevilla, Sevilla, Spain*

[6]*Department of Biological Chemistry, University of California, Los Angeles, Howard Hughes Medical Institute, CA, USA.*

[7]*Department of Pathology and Laboratory Medicine, Indiana University School of Medicine, Indianapolis, IN, USA.*

** Correspondence to: Lorena Saelices Gómez, lorena.saelicesgomez@utsouthwestern.edu*

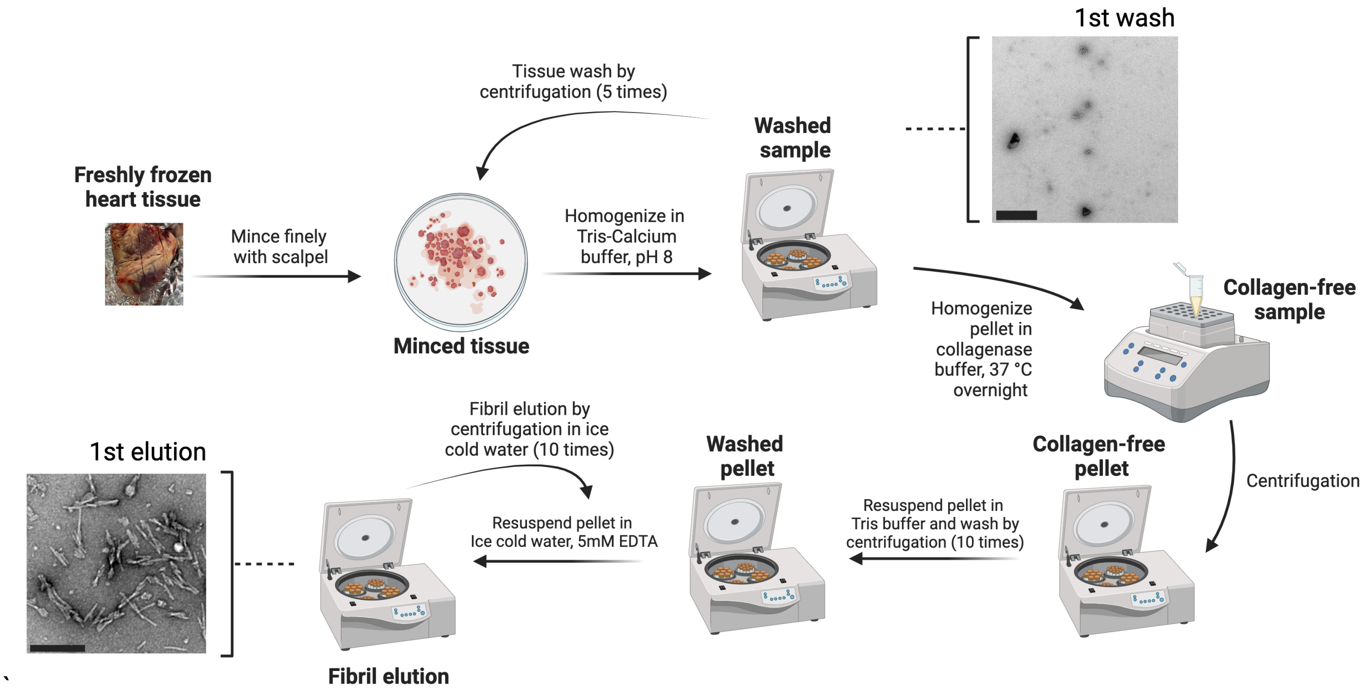

Supplementary Figure 1. Extraction and assessment of ATTRv-I84S fibrils from cardiac tissue. Schematic workflow of fibril extraction from human heart tissue, including a representative picture of a specimen. Representative negative stained TEM images of the first wash step of fibril extraction and the first fibril elution are included. Scale bar, 50 nm. Schemes are generated on BioRender.

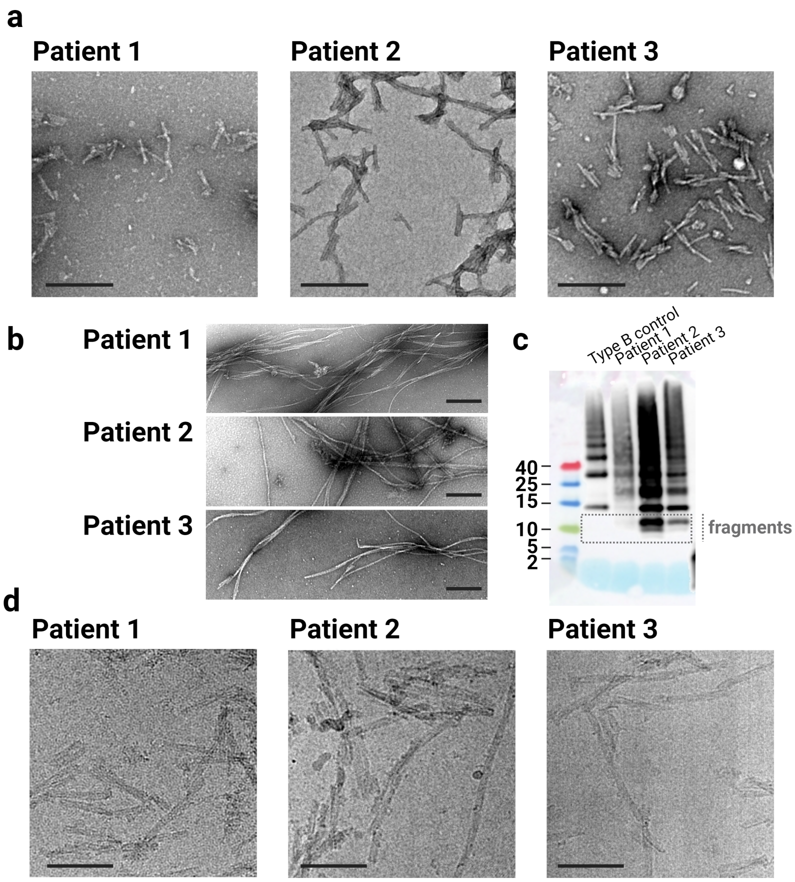

Supplementary Figure 2. Validation of fibril extraction from the heart of three ATTRv-I84S patients. a Negative stained images of extracted fibrils. Scale bar, 200 nm. **b** Negative stained images of recombinant fibrils from monomeric TTR after addition of ATTRv-I84S seeds at pH 7.4. **c** Anti-TTR western blot of extracted cardiac fibrils (0.5 µg) using an antibody that detect transthyretin fragmentation of the C-terminus. This assay is used to confirm that the analyzed ATTRv-I84S patients are type A. We use a Type B patient (Lane 1) as a control. All fibril samples extracted from ATTRv-I84S patients 1, 2, 3 (lanes 2-4) show transthyretin fragments characteristic of Type A. Dashed rectangle marks the C-terminus fragment region. **d** Representative cryo-EM micrographs of cardiac ATTRv-I84S fibrils from the three patients. Scale bar, 50 nm.

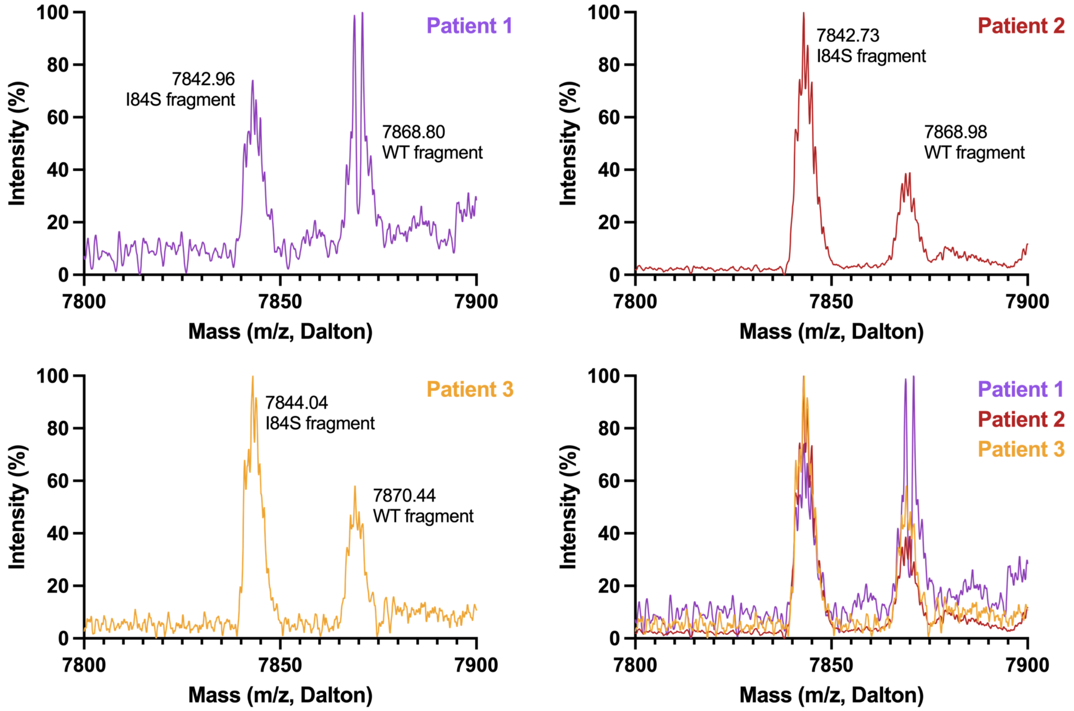

Supplementary Figure 3. Detection of intact C-terminal fragment Leu 58 to Glu 127 in fibril extracts. We performed intact mass spectrometry analysis to detect the presence of both wild-type (WT) and I84S peptide fragments (comprising residue Leu 58 to Glu 127) in fibrils extracts from the three ATTRv-I84S patients. Graphs show the observed neutral mass (m/z) with intensity profile for peptide representing WT fragments (7869.78 Da) and I84S fragments (7843.70 Da; loss of 26 Da for Ile to Ser mutation), deconvoluted from the raw data by mass spectrometry.

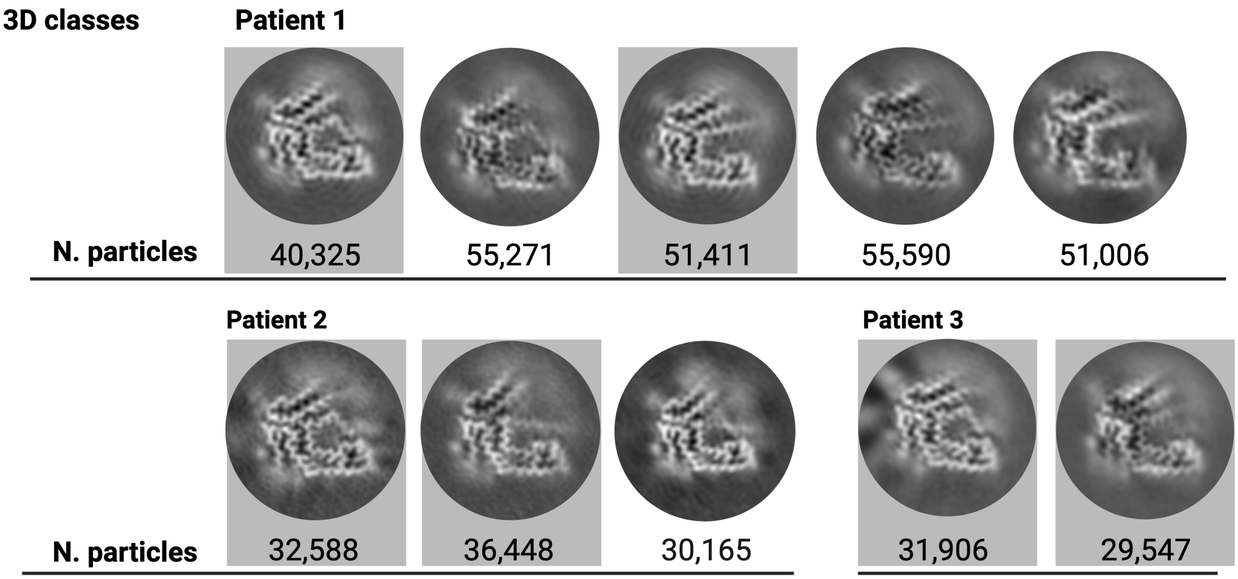

Supplementary Figure 4. 3D classification of ATTRv-I84S fibrils. 3D classification of fibril particles resulted in various distinct particle populations in each of the patients. Each patient featured a 3D class that resembled the structure previously observed in ATTR fibrils. Shaded maps are representative maps used in this paper.

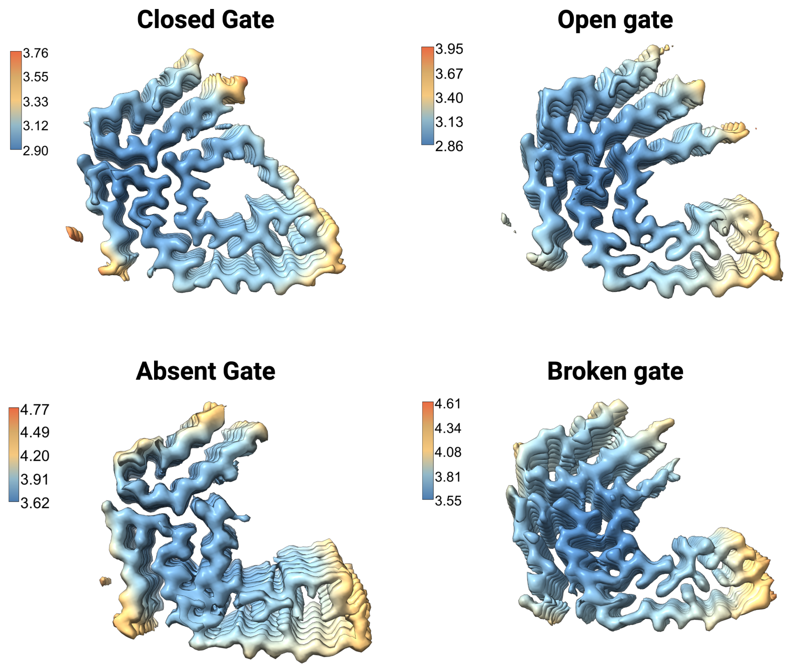

Supplementary Figure 5. Local resolution of ATTRv-I84S fibrils estimated using RELION colored as indicated on the final maps. Blue indicates higher resolution and red indicates lower resolution.

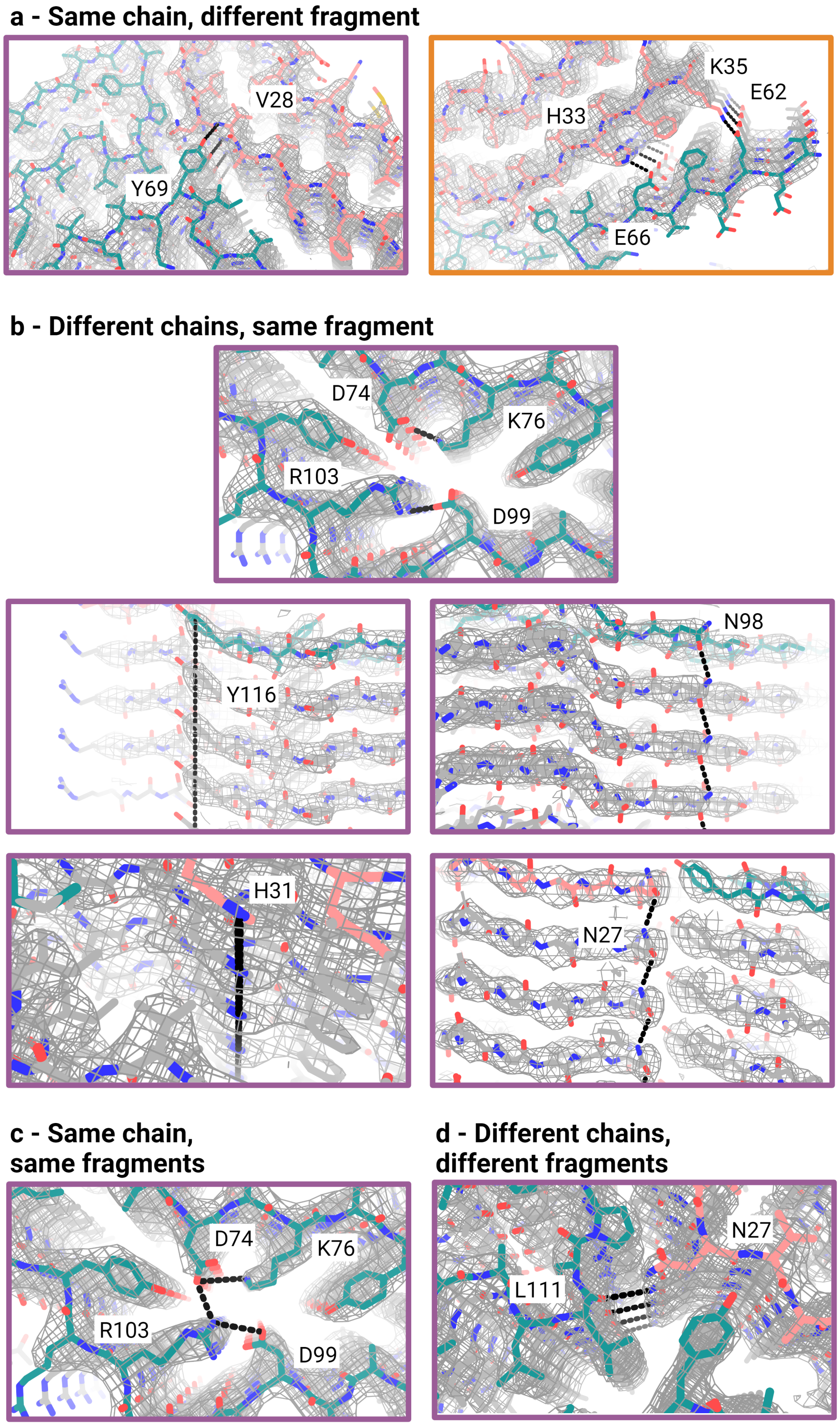

Supplementary Figure 6. Interactions within the ATTRv-I84S fibril structures that contribute to its stability and differ between the two fibril structures obtained in patient 1. This figure shows examples of interactions found in all the structures (here we use closed gate fold fibrils from patient 1, framed in purple rectangles) and the open gate fold exclusively (framed in an orange rectangle). **a** Interactions between residues from the same chain and different fragments, such as hydrophobic interactions between Val 28 and Tyr 69 (in

all structures), and salt bridges between Lys35 and Glu62, and between His33 and Glu66 (in the open gate fold). **b** Interactions between residues from different chains but the same fragment. These include, (i) π-π stacking neutralizing the ionizable side chains exposed to the outside (here we show Tyr 116) or to the interface between the C- and N- termini (here we show His 31); (ii) hydrogen bonding between the same residues stacking along the fibril (here we show Asn 98 and Asn 27); and (iii) salt bridges between multiple residues involving residues Asp 74, Lys 76, Asp 99 and Arg 103 from multiple layers. **c** Interactions between residues from the same chain and same fragments. **d** Interactions between residues from different chains and different fragments. One chain (or layer) is colored dark sea green as a reference. All interactions are noted as black dashed lines. Hydrogen bonding distance was between 2.7 Å and 3.4 Å. π-π stacking distance was between 4.8 Å and 4.9 Å.

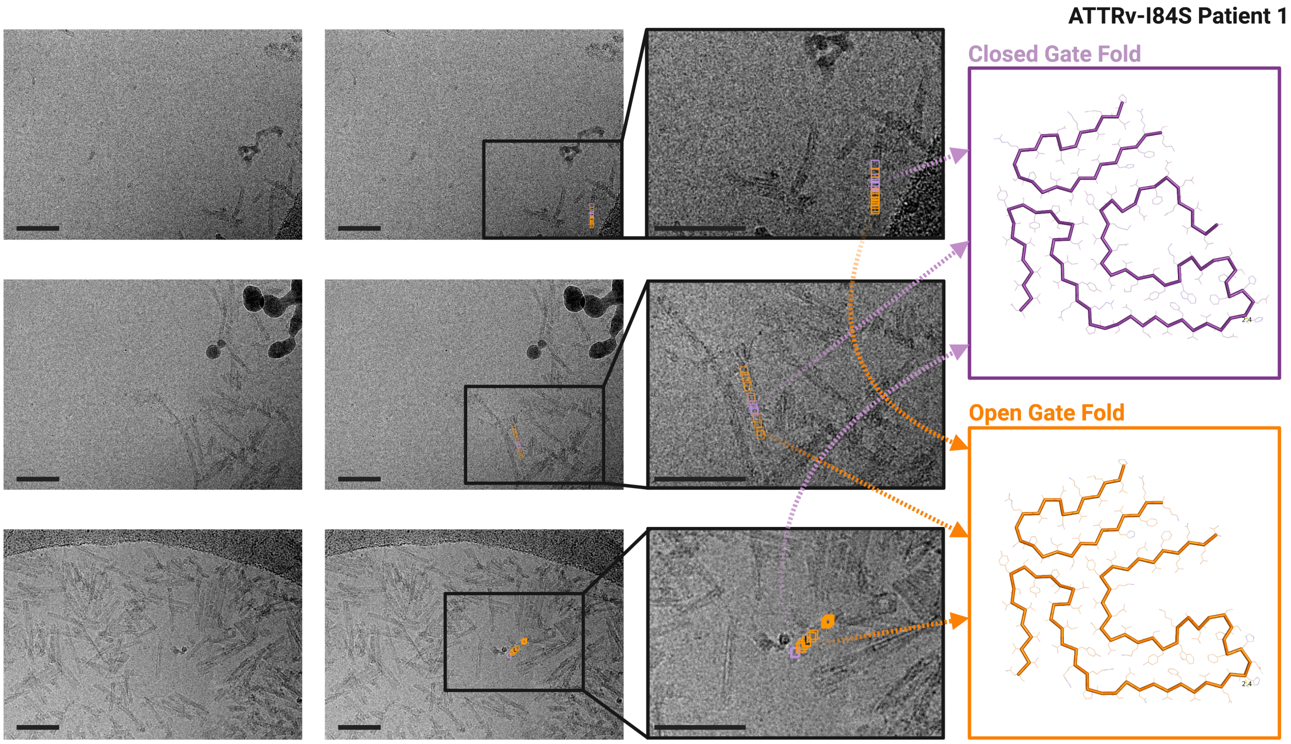

Supplementary Figure 7. **Evidence for the presence of different morphologies within the same fibril in ATTRv-I84S Patient 1.** Representative cryo-EM micrograph tracing the closed gate (purple) and the open gate (orange) folds back to the same fibril. Scale bar, 50 nm. The middle set of three figures are also included in article Figure 1k. Fifteen individual micrographs were used; the representing figure is from one micrograph.

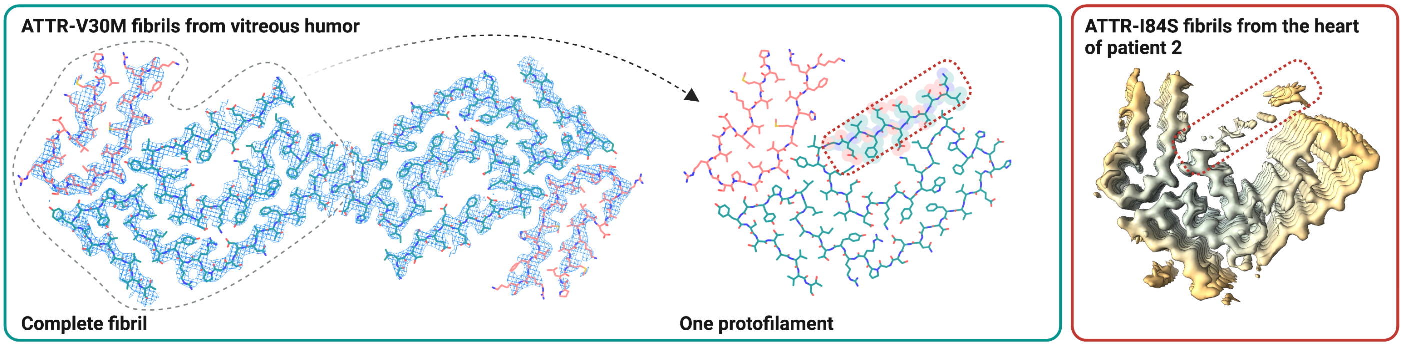

Supplementary Figure 8. ATTRv-V30M fibrils from vitreous humor (left) compared to the density map of ATTRv-I84S fibrils from the heart of Patient 2. Dashed rectangle marks residues involved in the polar pocket gate. Vitreous humor structure obtained from PDB 7OB4 [https://www.rcsb.org/structure/7OB4].

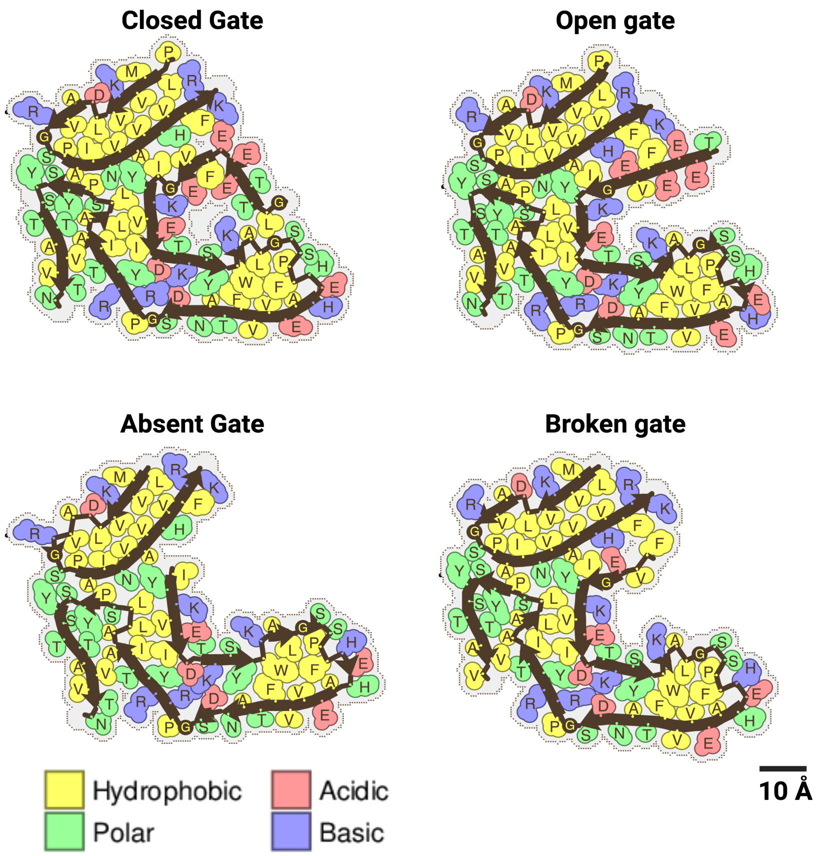

Supplementary Figure 9. Schematic view of ATTRv-I84S fibrils showing residue composition. Residues are color coded by amino acid category, as labeled.

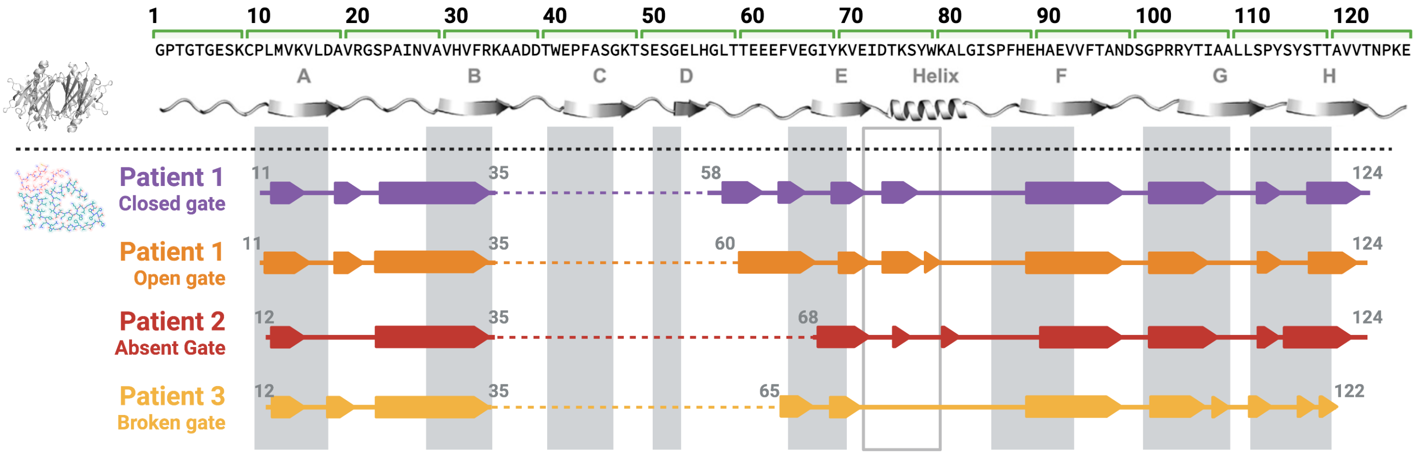

Supplementary Figure 10. Secondary structure composition of ATTRv-I84S fibrils. Top, transthyretin sequence and residue numbering. Below sequence and above dashed line, schematic representation of the secondary structure of native transthyretin, based on the crystal structure of native wild-type transthyretin (PDB 4TLT) [https://www.rcsb.org/structure/4TLT]. Letters label β-strands in the native structure. Below dashed line, secondary structure representation of fibrils from ATTRv-I84S patients. Arrows represent β-strands. Various fibril structures are color coded by patients.

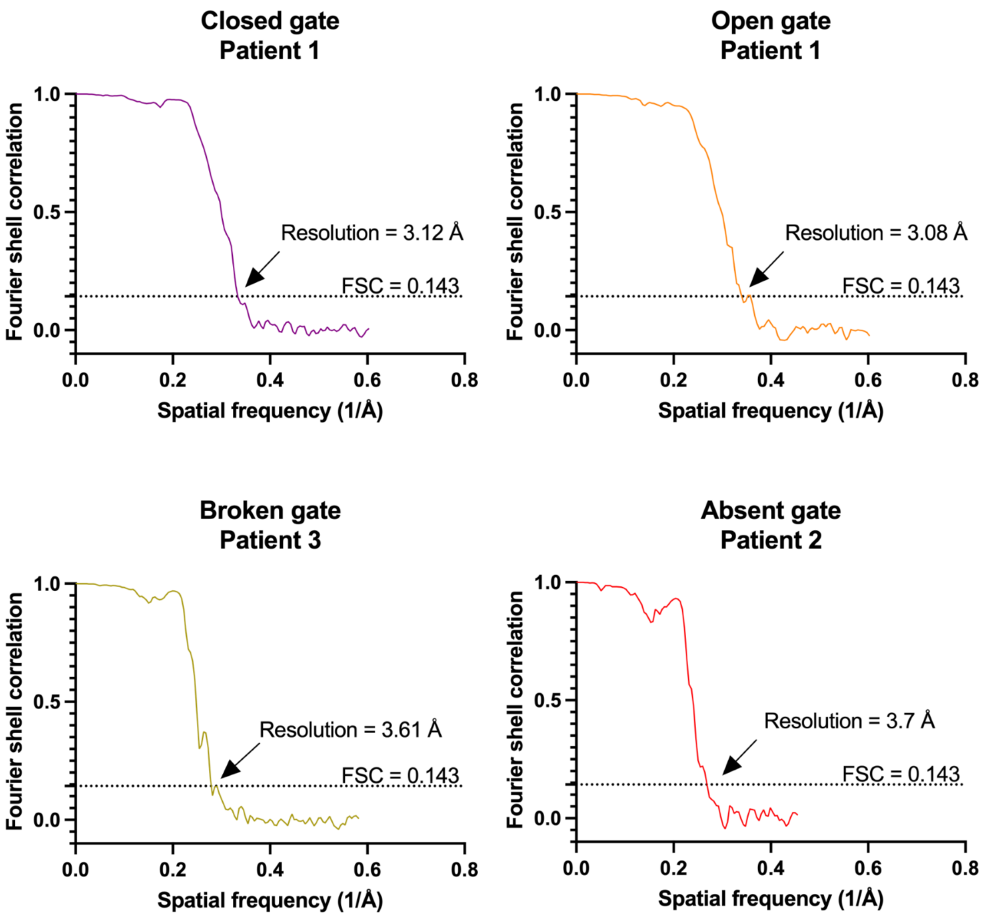

Supplementary Figure 11. FSC curves. Evaluation of the resolution of cryo-EM maps by Fourier shell correlation (FSC) curves of two independently refined half-maps from the ATTRv-I84S fibril structures of the three patients.

Supplementary Table 1. Identification of ATTR C-terminal fragment Leu 58 to Glu 127 using intact protein LC/MS.

	Observed neutral mass (Da)				
**Peak**	**Patient 1**	**Patient 2**	**Patient 3**	**TTR Sequence assignment**	**Theoretical Mass (Da)**
**Wild-type fragment**	7868.80	7868.98	7870.44	Leu58-Glu127	7869.78
**I84S fragment**	7842.96	7842.73	7844.04	Leu58-Glu127	7843.70

Supplementary Table 2. Species present in the three ATTRv-I84S patients and one wild-type (WT) control. Peptides represent both tryptic and semi-tryptic fragments.

**Peptide**		**ATTRv-I84S**			
	**TTR Sequence assignment**	**Patient 1**	**Patient 2**	**Patient 3**	**WT (Control)**
[A].GPTGTGESK.[C]	[1-9]	No	No	No	Yes
[K].CPLMVKVLDAVR.[G]	[10-21]	Yes	Yes	Yes	Yes
[K].VLDAVRGSPAINVAVHVFR.[K]	[16-34]	Yes	Yes	Yes	Yes
[R].GSPAINVAVHVFR.[K]	[22-34]	Yes	Yes	Yes	Yes
[R].GSPAINVAVHVFRKAADDTWEPFASGK.[T]	[22-48]	Yes	Yes	Yes	Yes
[K].VLDAVRGSPAINVAVHVFRKAADDTWEPF.[A]	[26-44]	Yes	Yes	Yes	Yes
[R].KAADDTWEPFASGK.[T]	[35-48]	Yes	Yes	Yes	Yes
[K].AADDTWEPFASGK.[T]	[36-48]	Yes	Yes	Yes	Yes
[P].FASGKTSESGELHGLTTEEEFVEGIYK.[V]	[44-70]	Yes	Yes	Yes	Yes
[G].LTTEEEFVEGIYK.[V]	[58-70]	Yes	Yes	Yes	Yes
[F].VEGIYKVEIDTK.[S]	[65-76]	Yes	Yes	Yes	Yes
[K].SYWKALGISPFHEHAEVVFTANDSGPR.[R]	[77-103]	Yes	Yes	Yes	Yes
[K].SYWKALG**S**SPFHEHAEVVFTANDSGPR.[R]	[77-103]	Yes	Yes	Yes	No
[K].ALGISPFHEHAEVVFTANDSGPR.[R]	[81-103]	Yes	Yes	Yes	Yes
[R].RYTIAALLSPYSYSTTAVVTNPKE.[-]	[104-127]	Yes	Yes	Yes	Yes
[L].SPYSYSTTAVVTNPKE.[-]	[112-127]	Yes	Yes	Yes	Yes

*Mutation Ile to Ser highlighted in red (Bold) in fragment 77-103.

Supplementary Table 3: Data collection and refinement statistics.

Data collection	ATTRv-I84S Patient 1 Closed gate	ATTRv-I84S Patient 1 Open gate	ATTRv-I84S Patient 2 Absent gate	ATTRv-I84S Patient 3 Broken gate
**Microscope**	Titan Krios	Titan Krios	Titan Krios (G3i)	Titan Krios (G3i)
**Acceleration Voltage (kV)**	300	300	300	300
**Detector**	K3	K3	K3	K3
**Software**	SerialEM 3.8	SerialEM 3.8	EPU	EPU
**Magnification**	105,000x	105,000x	81,000x	105,000x
**Pixel size at detector (Å/pixel)**	0.83	0.83	1.1	0.86
**Defocus range (µm)**	-0.9 to -2.2	-0.9 to -2.2	-1.5 to -2.1	-0.8 to -2.1
**Total dose (e^-/Å^2)**	60	60	50	50
**Exposure time (s)**	5.4	5.4	3	1.81
**Number of movie frames**	60	60	33	40
**Usable micrograph**	6004	6004	7223	9266
**Box size (pixel)**	256	256	256	256
**Total extracted segments**	1066249	1066249	985994	475685
**Number of segments after 2D (curvy)**	609395	609395	328559	344855
**Number of straight segments (2D)**	194978	194978	218585	43762
**Number of segments after 3D**	40325	51411	36448	29547
**Symmetry imposed**	C1	C1	C1	C1
**Helical rise (Å)**	4.81	4.80	4.93	4.96
**Helical twist (°)**	-1.26	-1.42	-1.30	-1.33
**Crossover length (Å)**	689	608	683	672
**B factor**	-79.23	-93.5	-104	-106
**Map resolution (Å; FSC=0.143)**	3.1	3.1	3.8	3.6
**Map resolution (Å; FSC=0.5)**	3.4	3.4	4.2	4.1
**Non-hydrogen atoms**	3620	2892	3165	3245
**Protein residues**	465	372	405	415
**Number of chains**	5	4	5	5
**Water/ligands**	0/0	0/0	0/0	0/0
**MolProbity score**	1.99	1.93	1.99	1.88
**Clash score**	9.57	16.36	11.18	8.92
**Rotamer outliers (%)**	0.00	0.00	0.58	0.86
**R.M.S deviations bonds (Å)**	0.004	0.005	0.004	0.004
**R.M.S deviations angle (°)**	0.54	0.556	0.727	0.671
**Ramachandran plot**				
Favored	92.12	96.63	93.51	93.92
Allowed	7.87	3.37	6.49	6.08
Outliers	0.00	0.00	0.00	0.00
**CaBLAM outliers (%)**	2.35	4.71	1.37	6.13
**Model vs Data**	0.86	0.84	0.8	0.8
