# Supplementary material for: Structural polymorphism of amyloid fibrils in ATTR amyloidosis revealed by cryo-electron microscopy
Source: Nat Commun. 2024 Jan 17;15:581. doi: 10.1038/s41467-024-44820-3 (PMC10794703; doi:10.1038/s41467-024-44820-3)
Supplement: Supplementary file 3 — Reporting Summary [file 41467_2024_44820_MOESM3_ESM.pdf]

nature portfolio

Corresponding author(s): Lorena Saelices

Last updated by author(s): 2023/12/07

Reporting Summary

Nature Portfolio wishes to improve the reproducibility of the work that we publish. This form provides structure for consistency and transparency in reporting. For further information on Nature Portfolio policies, see our Editorial Policies and the Editorial Policy Checklist.

Statistics

For all statistical analyses, confirm that the following items are present in the figure legend, table legend, main text, or Methods section.

n/a	Confirmed	
☐	☒	The exact sample size (*n*) for each experimental group/condition, given as a discrete number and unit of measurement
☐	☒	A statement on whether measurements were taken from distinct samples or whether the same sample was measured repeatedly
☐	☒	The statistical test(s) used AND whether they are one- or two-sided<br>*Only common tests should be described solely by name; describe more complex techniques in the Methods section.*
☒	☐	A description of all covariates tested
☒	☐	A description of any assumptions or corrections, such as tests of normality and adjustment for multiple comparisons
☐	☒	A full description of the statistical parameters including central tendency (e.g. means) or other basic estimates (e.g. regression coefficient) AND variation (e.g. standard deviation) or associated estimates of uncertainty (e.g. confidence intervals)
☐	☒	For null hypothesis testing, the test statistic (e.g. *F*, *t*, *r*) with confidence intervals, effect sizes, degrees of freedom and *P* value noted<br>*Give P values as exact values whenever suitable.*
☒	☐	For Bayesian analysis, information on the choice of priors and Markov chain Monte Carlo settings
☒	☐	For hierarchical and complex designs, identification of the appropriate level for tests and full reporting of outcomes
☒	☐	Estimates of effect sizes (e.g. Cohen's *d*, Pearson's *r*), indicating how they were calculated

Our web collection on statistics for biologists contains articles on many of the points above.

Software and code

Policy information about availability of computer code

Data collection	EPU and SerialEM
Data analysis	Relion 4.0 (MRC, LMB Cambridge), CTFFIND4.1 (Rohou, A. & Grigorieff), COOT (Emsley et al., 2010) and PHENIX (Afonine et al., 2018 for Cryo-EM and graphPad prism 9.0 (for Mac) for rest of the analysis, Biorender for diagrams, EMAN2 (Tang et al., 2007)

For manuscripts utilizing custom algorithms or software that are central to the research but not yet described in published literature, software must be made available to editors and reviewers. We strongly encourage code deposition in a community repository (e.g. GitHub). See the Nature Portfolio guidelines for submitting code & software for further information.

Data

Policy information about availability of data

All manuscripts must include a data availability statement. This statement should provide the following information, where applicable:

- Accession codes, unique identifiers, or web links for publicly available datasets
- A description of any restrictions on data availability
- For clinical datasets or third party data, please ensure that the statement adheres to our policy

Structural data have been deposited into the Worldwide Protein Data Bank (wwPDB) and the Electron Microscopy Data Bank (EMDB) with the following EMD accession codes: 41171 (Closed Gate, Patient 1) [https://www.ebi.ac.uk/emdb/EMD-41171], 41172 (Open Gate) [https://www.ebi.ac.uk/emdb/EMD-41172], 26685 (Absent Gate) [https://www.ebi.ac.uk/emdb/EMD-26685], 27323 (Broken Gate) [https://www.ebi.ac.uk/emdb/EMD-27323], and PDB accession codes: 8TDN

(Closed Gate, Patient 1) [https://www.rcsb.org/structure/8TDN], 8TDO (Open Gate) [https://www.rcsb.org/structure/8TDO], 8E7E (Absent Gate) [https://www.rcsb.org/structure/8E7E], 8E7J (Broken Gate) [https://www.rcsb.org/structure/8E7J]. The PDB accession codes for the previously reported coordinates of ATTRv-V30M fibrils from vitreous humor and heart are 7OB4 [https://www.rcsb.org/structure/7OB4] and 6SDZ [https://www.rcsb.org/structure/6SDZ], respectively. All data generated or analyzed during this study that support the findings are available within this published article and its supplementary data files. MS Data are available via MassIVE (a member of ProteomeXchange) with identifier MSV000093061 [https://massive.ucsd.edu/ProteoSAFe/dataset.jsp?task=019f119d13b747d09e22bc352e41b7d6] for QTOF data and MSV000093062 [https://massive.ucsd.edu/ProteoSAFe/dataset.jsp?task=3f0caffc5cb74a1f85e5b9e6ce8a8b5a] for LC-MS/MS data. Graphed data is provided in the source data file.

Human research participants

Policy information about studies involving human research participants and Sex and Gender in Research.

Reporting on sex and gender	Due to the limited sample availability a sex based analysis of the sample was not performed.
Population characteristics	We obtained fresh frozen and lyophilized cardiac tissues from ATTR patients carrying TTR mutations I84S (n=3), Fresh frozen tissue for Type B V30M (n=1) and Wild type (n=1)
Recruitment	Specimens from the left ventricle of either explanted or autopsied hearts were obtained from the laboratory of late Dr. Merrill D. Benson at the University of Indiana.
Ethics oversight	The Office of the Human Research Protection Program granted exemption from Internal Review Board review because all specimens were anonymized.

Note that full information on the approval of the study protocol must also be provided in the manuscript.

Field-specific reporting

Please select the one below that is the best fit for your research. If you are not sure, read the appropriate sections before making your selection.

☒ Life sciences ☐ Behavioural & social sciences ☐ Ecological, evolutionary & environmental sciences

For a reference copy of the document with all sections, see nature.com/documents/nr-reporting-summary-flat.pdf

Life sciences study design

All studies must disclose on these points even when the disclosure is negative.

Sample size	Based on our experience, 5000 micrographs (collected under the parameters defined in supplementary table 3) are sufficient for high resolution reconstructions. Therefore, the sample size was deemed sufficient based on the quality of the final reconstructions and the resolution range achieved (3.1 to 3.8 A).
Data exclusions	Particles with poor SNR each data-set in 2D classification were excluded from the final 3D reconstructions. Moreover, straight filaments with no twist that cannot be resolved due to the current technical limitations were excluded from the sample to ensure sample homogeneity.
Replication	We performed 5 reconstructions for each dataset and observed the same results, thus confirming the reproducibility of these results.
Randomization	For the current design of the study, randomization was not relevant because this was a single case study.
Blinding	Particles (segments) were picked manually by several authors to avoid bias in particle picking. For the downstream analysis, we used softwares (Relion 4.0) to avoid any potential bias. Since the sample contained only ATTR amyloid fibrils no blinding was required during fibril extraction and purification steps.

Reporting for specific materials, systems and methods

We require information from authors about some types of materials, experimental systems and methods used in many studies. Here, indicate whether each material, system or method listed is relevant to your study. If you are not sure if a list item applies to your research, read the appropriate section before selecting a response.

Materials & experimental systems

n/a	Involved in the study
☐	☒ Antibodies
☒	☐ Eukaryotic cell lines
☒	☐ Palaeontology and archaeology
☒	☐ Animals and other organisms
☒	☐ Clinical data
☒	☐ Dual use research of concern

Methods

n/a	Involved in the study
☒	☐ ChIP-seq
☒	☐ Flow cytometry
☒	☐ MRI-based neuroimaging

Antibodies

Antibodies used

Primary rabbit, polyclonal antibody against the C-terminus of human transthyretin sequence (1;1000), synthesized by genscript.
Secondary antibody Goat anti-rabbit IgG (H+L) Secondary Antibody, HRP(1:1000) from Invitrogen was used as a secondary antibody.

Validation

Primary rabbit, polyclonal antibody against the C-terminus of human transthyretin sequence: Antibody was validated by Genscript using indirect ELISA. Additionally, we tested binding to the target peptide sequence and to confirmed transthyretin containing samples (Purified recombinant and amyloid extracts)
Goat anti-rabbit IgG (H+L) Secondary Antibody, HRP(1:1000) from Invitrogen: According to the manufacturers, the antibody has been validated for use in western blots, IHC and IP applications
