# Supplementary material for: Structural polymorphism of amyloid fibrils in ATTR amyloidosis revealed by cryo-electron microscopy
Source: Nat Commun. 2024 Jan 17;15:581. doi: 10.1038/s41467-024-44820-3 (PMC10794703; doi:10.1038/s41467-024-44820-3)
Supplement: Supplementary file 2 — Peer Review File [file 41467_2024_44820_MOESM2_ESM.pdf]

nature portfolio

Peer Review File

Structural polymorphism of amyloid fibrils in ATTR amyloidosis revealed by cryo-electron microscopy

Open Access This file is licensed under a Creative Commons Attribution 4.0 International License, which permits use, sharing, adaptation, distribution and reproduction in any medium or format, as long as you give appropriate credit to the original author(s) and the source, provide a link to the Creative Commons license, and indicate if changes were made. In the cases where the authors are anonymous, such as is the case for the reports of anonymous peer reviewers, author attribution should be to 'Anonymous Referee' followed by a clear attribution to the source work. The images or other third party material in this file are included in the article's Creative Commons license, unless indicated otherwise in a credit line to the material. If material is not included in the article's Creative Commons license and your intended use is not permitted by statutory regulation or exceeds the permitted use, you will need to obtain permission directly from the copyright holder. To view a copy of this license, visit http://creativecommons.org/licenses/by/4.0/.

REVIEWER COMMENTS

Reviewer #1 (Remarks to the Author):

This is a very interesting manuscript describing amyloid fibril polymorphism in ATTR amyloidosis. The findings are novel and will be of high interest to the amyloid research community. The research has been conducted at a very high technical level. The conclusions drawn by the authors are important, and in general, very well supported by their data.

When reading the manuscript several questions came to mind that I would like the authors to consider.

Main questions

1. The finding of straight fibrils from ATTRv-I84S patient 1 is very interesting. Can the authors provide an estimate of what proportion of total fibrils were straight. Straight fibrils were apparently not seen in patients 2 and 3 and therefore their presence in heart tissue in patient 1 does not appear to influence a consistent clinicopathological phenotype. It would be helpful if this point could be elaborated.

2. With regard to co-existence of different polymorphs within the same fibril it would be extremely valuable to know about proportions and whether all fibrils were mixed or if there was evidence of fibrils that were composed solely of either wild-type or mutant TTR. The images as presented in Fig 1k and SI Fig 4 are not overwhelmingly convincing as they show minimal interspersed segments. Were there individual fibrils containing longer stretches of adjacent segments of each polymorph? If so, these should be shown.

3. It would be helpful if the mass spectrometry methods were expanded to include more detail of how the tryptic peptides encompassing variant residue 84 (containing either I or S) were quantified. This quantitation can be complex as the two peptides may not ionise with the same efficiency. Were synthetic peptides used for calibration? The percentage of wild-type peptide in patient 1 was quite dissimilar to patients 2 and 3, could the straight fibrils account for this?

4. Can the authors expand Supplementary Table 1 as in its current form it is not very informative. In this regard the authors discuss a consistent clinical phenotype and it would be useful to include any available clinical histories. Also, why is the age of patient 1 an estimate?

Reviewer #2 (Remarks to the Author):

The manuscript “Structural polymorphism of amyloid fibrils in ATTR amyloidosis revealed by cryo-electron microscopy” by Nguyen et al describes the ex vivo structures of the amyloid deposits found in three ATTR patients carrying the same amyloidogenic TTR mutation (I84S). The manuscript reports relevant scientific data but their interpretation is skewed and the take home message of the manuscript is not supported by the structures. In the present form this manuscript cannot be published.

Main comment:

The authors structurally characterise the amyloid fibrils extracted from three ATTR patients carrying the same mutation. In all cases they found the same structure which they name closed gate, however given

that it is always the same they determined and discussed it for patient 1 while they fully ignore it in the other two patients. Moreover, judging from the figures shown in the manuscript it seems to me that the closed gate structure is the highest quality 2D class also in patient 2 and 3. Even though this closed gate structure is always present, they barely mention it and never show it in the figures.

In their other structures, about 80-90% of the residues form the usual TTR amyloid fold which is common to the closed gate structure and to all other TTR structures so far reported. The polypeptide stretch they find in different conformations is a solvent exposed polypeptide segment which in some case is found in different conformations and sometimes is simply invisible (maybe it is simply proteolysed?). In any case let's keep in mind that structural biology is full of examples showing that even two structures from the very same sample may not be identical, this may be especially true for ex-vivo structures where different part of the tissue may have slightly different physio-pathological conditions and amyloid deposits in different patients may have significantly different age.

In summary, the authors overinterpret these minor differences as to claim that different patients have different structures and even challenge the idea of one disease associates with one fibril fold proposed after the structural data obtained on A-beta and tau fibrils. These claims are baseless as the structural data point exactly in the opposite direction.

Structural data instead show that the I84S mutation partially destabilises the closed gate conformation leading to a mixture of conformers, but it also shows the resilience of TTR amyloid fold.

Thus, the title, abstract, presentation of the data, discussion and take-home message must be completely revised and made consistent with the experimental data.

Here some major and minor comments:

- the estimated energy of salvation shows that all fibrils are based on the same stabilising core with the channel giving a minor contribution. That's why the channel region can be found in different conformations.

- Fig 5 and Fig S8 are misleading, the closed structure is present in all three patients but the authors are not showing them here or elsewhere. It is crucial to mention and show them all over.

- pg 13 contrast to one fibril one disease: it is highly speculative, this polymorphs show simply that the I84S destabilise the internal gate of TTR fibrils. Moreover the seem to forget here that they have the same closed conformation in all patients.

- paragraph pg 14 is very speculative: if the different conformations seen in patients are affected or affect proteolysis, this should be checked by MS of fibrils.

- The claim that the content of the cavity may play a role in pathology is way too speculative

- electrophoretic and MS analysis of the fibrils would be very useful to provide explanations to the different conformation found in the three patients.

Here some minor comments:

- It is very burdensome to have supplementary figures embedded in the main text, please remove the supplementary figures from the main text.

- supplementary figure 3 the percentages are wrong as they do not take in account the particles which are not used for 3D reconstruction

- page 10 correct “mayor” with “major”

- pg 10 the missing region in the fibrils is not folded as coiled coil in the native structure but it is a beta strand - loop - beta edge strand.

- when rmsd is calculated it is never stated on how many Ca

- Fig. 1E and H, 2E and 3E: the resolution range goes to 5.6 Å but the resolution seems to be very homogeneous, my best guess is that there is a problem in the calculation of the resolution or else the color range should modified to appreciate the local differences in resolution.

Reviewer #3 (Remarks to the Author):

This is a potentially important paper addressing the role of amyloid polymorphs in disease phenotypes. The authors determined the structures of cardiac fibrils extracted from three ATTR amyloidosis patients all carrying the ATTRv-I84S mutation. They found multiple ATTR structures with structural variations specific to the individual. They argue that current results question the association between phenotype and fibril structure. The experiments were carefully done, and the elucidated amyloid structures are interesting. Although this reviewer is not familiar with the details of cryo-EM analysis, the results seem basically valid. However, there are some concerns to be clarified.

1. Regarding the proposal that “this finding challenges the paradigm of one amyloid disease associates with one fibril fold. I recall Strohaker, T., et al. (2019). "Structural heterogeneity of alpha-synuclein fibrils amplified from patient brain extracts." Nat Commun 10(1): 5535, in which the authors raised questions regarding the one disease-one strain hypothesis. Similar questions have been raised in several papers [https://doi.org/10.1038/s41586-020-2317-6; DOI: 10.1002/anie.201511524; https://doi.org/10.1038/s41467-019-13564-w]. The authors should clarify the uniqueness of this paper in comparison with those related papers.

2. Figure 6. A schematic representation of the fibril polymorphism observed in ATTR fibrils: Although polymorphs shown in Fig. 6 are distinct in detail, they are similar to each other in the core structures. Recently, Wilkinson et al. (https://doi.org/10.1038/s41467-023-36791-8) reported a paper showing that a hammer-shaped motif makes up the core of all b2-microgobluin polymorphs. It seems that a common core structure is an important observation for understanding the mechanism of TTR amyloid formation, as well as polymorphs observed in non-core regions.

3. Sup. Fig. 2: “The analyzed ATTRv-I84S patients are type A with intact TTR and C-terminus TTR fragment”. Could the authors specify the residue numbers for the C-terminus fragment? Is that TTR50-127? From Sup. Fig. 2C lane 3, the fraction of the C-terminus fragment is higher than 50% of the total. For lane 3, the bands for the intact and fragment TTR are unclear. All CryoEM structures reported in this

paper show the entire regions although N-terminal (1-10) and 35-55 regions were missing (Fig. 4). Then, how do the intact and fragmented TTRs coexist in fibril structures determined in this paper?

4. In type A fibrils, when does the proteolytic cleavage occur? Is that after formation of amyloid fibrils, leaving the cleaved peptides in fibrils and making the structural analysis with the nicked N-terminal peptide possible? Probably, this is not the case.

5. Page 14 "The structural variability that we found in cardiac ATTR fibrils indicates that type A pathology can consist of structural polymorphs." Is it possible that proteolytic cleavage affects the CryoEM analysis leading to apparent structural polymorphs?

6. Fig. 1K. Sup. Fig. 4. "Evidence for the presence of different morphologies within the same fibril". This observation is amazing although the validity should be carefully confirmed. The authors established seeding method for amplifying amyloid fibrils purified from patients (Sup. Fig. 2B). Do the different morphologies within the same fibril propagate during in vitro seeding? Or is it a rare phenomenon only occurring in vivo?

7. Fig. 5. "Fibril stability in ATTRv-I84S amyloidosis fibrils". "ATTR fibrils on average are estimated significantly more stable than other fibrils, both by residue and chain (Figure 5A and 5B)". Experimental evidence supporting this argument will be necessary.

Reviewer 1

<u>Reviewer 1, comment 1.</u> *The finding of straight fibrils from ATTRv-I84S patient 1 is very interesting. Can the authors provide an estimate of what proportion of total fibrils were straight. Straight fibrils were apparently not seen in patients 2 and 3 and therefore their presence in heart tissue in patient1 does not appear to influence a consistent clinicopathological phenotype. It would be helpful if this point could be elaborated.*

Response: We thank the reviewers for pointing this out. We do observe straight fibrils in all three patients. We are unsure of the relevance of these straight fibrils to the phenotype at the moment. In response to the reviewer, we added additional information to the Supplementary Table 3 to share the number of straight and twisted fibrils in all three cases. We also incorporated them into the manuscript text under the results section with the heading "ATTRv-I84S fibrils are structurally polymorphic." as follows: "The straight species was not suitable for structure determination by helical reconstruction because it lacked a twist (Fig. 1a and Supplementary Table 3) on page 3, lines 13-14.

<u>Reviewer 1, comment 2.</u> *With regard to co-existence of different polymorphs within the same fibril it would be extremely valuable to know about proportions and whether all fibrils were mixed or if there was evidence of fibrils that were composed solely of either wild-type or mutant TTR. The images as presented in Fig 1k and SI Fig 4 are not overwhelmingly convincing as they show minimal interspersed segments. Were there individual fibrils containing longer stretches of adjacent segments of each polymorph? If so, these should be shown.*

Response: In the process of reconstructing the fibrils using RELION, coordinates of fibrils were initially segmented during the Extraction step into smaller particles. These particles then underwent multiple rounds of 2D and 3D classifications to identify the highest-quality ones. These particles were subsequently selected and averaged to create the final fibril map. As a result of this selection process, often only a limited number of well-defined particles from the same fibril were included i.e. bad particles on the same fibril were excluded. Therefore, fibril particle images may appear as interspersed segments on the same fibrils. We have also seen a large number of fibrils containing particles of either wild-type or mutant TTR. These particles, however, were also interspersed due to the same reason explained above.

To make the content clearer, we have modified this method in the manuscript (Page 13, Line 24-30) as follows: "**Filament tracing**. The coordinates of fibril segments (rlnCoordinateX and rlnCoordinateY) of the final Refine3D were extracted from the relion file run_data.star. These coordinates were plotted onto XY-graphs and the graphs were adjusted to match the corresponding micrographs dimensions. It is important to note that only the well-defined segments of fibrils were included for reconstruction, omitting any subpar segments. As a result, the positions of these well-defined segments, especially when they belong to the same fibril, may appear interspersed as illustrated in Fig. 1k and Supplementary Fig. 7."

Reviewer 1, comment 3. *It would be helpful if the mass spectrometry methods were expanded to include more detail of how the tryptic peptides encompassing variant residue 84 (containing either I or S) were quantified. This quantitation can be complex as the two peptides may not ionise with the same efficiency. Were synthetic peptides used for calibration? The percentage of wild-type peptide inpatient 1 was quite dissimilar to patients 2 and 3, could the straight fibrils account for this?*

Response: We thank the reviewer for pointing out the complexities associated with this analysis. We have now removed the quantification of the peptides because of a lack of a calibration system. We have performed additional mass spectrometry experiments to confirm genotype. On page 3, lines 3-6 we write "Additionally, using mass spectrometry, we showed that all fibril samples contain both wild-type and mutant transthyretin, confirming that all patients included in this study are heterozygous for the ATTRv-I84S mutation (Supplementary Table 1 and 2 and Supplementary Fig. 3)."

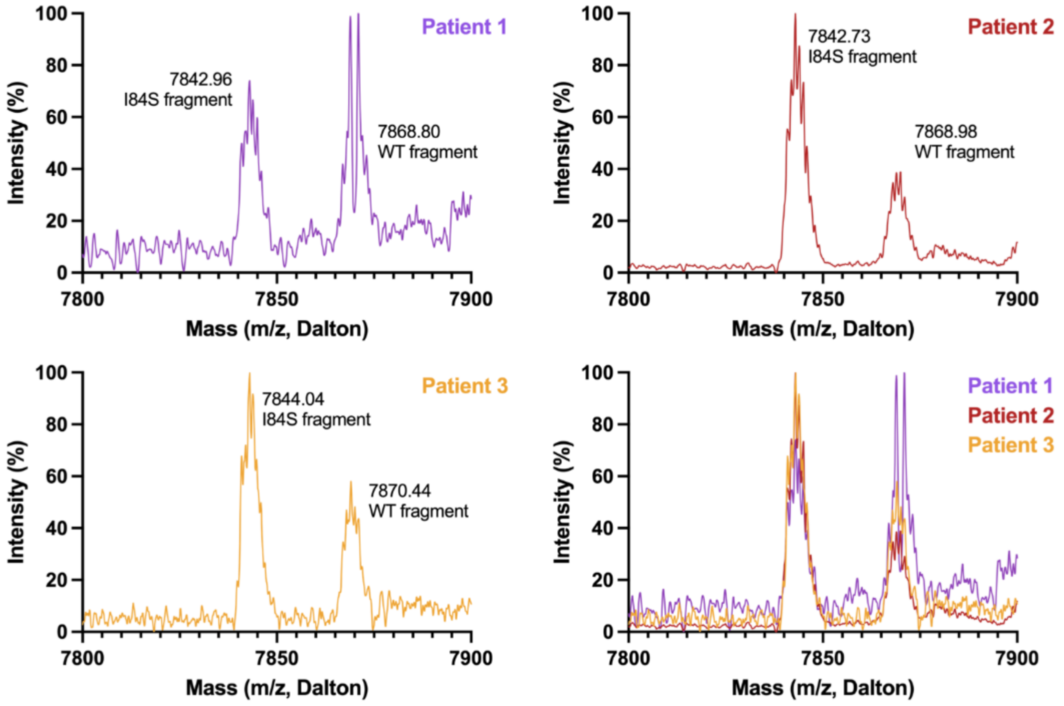

Supplementary Figure 3. Detection of intact C-terminal fragment Leu 58 to Glu 127 in fibril extracts. We performed intact mass spectrometry analysis to detect the presence of both wild-type (WT) and I84S peptide fragments (comprising residue Leu 58 to Glu 127) in fibrils extracts from the three ATTRv-I84S patients. Graphs show the observed neutral mass (m/z) with intensity profile for peptide representing WT fragments (7869.78 Da) and I84S fragments (7843.70 Da; loss of 26 Da for Ile to Ser mutation), deconvoluted from the raw data by mass spectrometry.

Supplementary Table 1. Identification of ATTR C-terminal fragment Leu 58 to Glu 127 using intact protein LC/MS.

	Observed neutral mass (Da)				
**Peak**	**Patient 1**	**Patient 2**	**Patient 3**	**TTR Sequence assignment**	**Theoretical Mass (Da)**
**Wild-type fragment**	7868.80	7868.98	7870.44	Leu58-Glu127	7869.78
**I84S fragment**	7842.96	7842.73	7844.04	Leu58-Glu127	7843.70

Supplementary Table 2. Species present in the three ATTRv-I84S patients and one wild-type (WT) control. Peptides represent both tryptic and semi-tryptic fragments.

**Peptide**		**ATTRv-I84S**			
	**TTR Sequence assignment**	**Patient 1**	**Patient 2**	**Patient 3**	**WT (Control)**
[A].GPTGTGESK.[C]	[1-9]	No	No	No	Yes
[K].CPLMVKVLDAVR.[G]	[10-21]	Yes	Yes	Yes	Yes
[K].VLDAVRGSPAINVAVHVFR.[K]	[16-34]	Yes	Yes	Yes	Yes
[R].GSPAINVAVHVFR.[K]	[22-34]	Yes	Yes	Yes	Yes
[R].GSPAINVAVHVFRKAADDTWEPFASGK.[T]	[22-48]	Yes	Yes	Yes	Yes
[K].VLDAVRGSPAINVAVHVFRKAADDTWEPF.[A]	[26-44]	Yes	Yes	Yes	Yes
[R].KAADDTWEPFASGK.[T]	[35-48]	Yes	Yes	Yes	Yes
[K].AADDTWEPFASGK.[T]	[36-48]	Yes	Yes	Yes	Yes
[P].FASGKTSESGELHGLTTEEEFVEGIYK.[V]	[44-70]	Yes	Yes	Yes	Yes
[G].LTTEEEFVEGIYK.[V]	[58-70]	Yes	Yes	Yes	Yes
[F].VEGIYKVEIDTK.[S]	[65-76]	Yes	Yes	Yes	Yes
[K].SYWKALGISPFHEHAEVVFTANDSGPR.[R]	[77-103]	Yes	Yes	Yes	Yes
[K].ALGISPFHEHAEVVFTANDSGPR.[R]	[81-103]	Yes	Yes	Yes	Yes
[R].RYTIAALLSPYSYSTTAVVTNPKE.[-]	[104-127]	Yes	Yes	Yes	Yes
[L].SPYSYSTTAVVTNPKE.[-]	[112-127]	Yes	Yes	Yes	Yes

Reviewer 1, comment 4 *Can the authors expand Supplementary Table 1 as in its current form it is not very informative. In this regard the authors discuss a consistent clinical phenotype and it would be useful to include any available clinical histories. Also, why is the age of patient 1 an estimate?*

Response: We agree with the reviewer's comment. The clinical history that we have from these patients is indeed limited. These samples were obtained from the late Dr. Benson months before he passed away, and most of his notes were in personal notebooks and his memories, which we do not have access to. Thus, we have removed the table and incorporated the limited information into *Patient and Tissue Materials* section in Methods, page 12, Lines 13-16 which now reads, "ATTRv-I84S patients 1 and 3 were male and patient 2 was a female. Patient with wild-type ATTR was 78 years male with transplanted heart. Patient with type B ATTR was male caring V30M TTR mutation with peripheral and autonomic neuropathy and died at the age of 42 years. All patients were in their 50's at the time of collection." We have also included a *Limitations* section in the Discussion that reads as follows: "Because of our limited access to the clinical histories' of these patients, correlations between disease progression and structure cannot be discerned. Additionally, in this present study, we have looked at heart samples from three patients with one mutation. Future studies that involve structural analysis of more mutations and other organs as well as experimental validation of their stability can shed further light on the role of mutations and influence of the tissue microenvironment in structural polymorphism in ATTR amyloidosis." Page 11, Lines 37-42.

Reviewer #2:

Reviewer 2, comment 1. *The authors structurally characterise the amyloid fibrils extracted from three ATTR patients carrying the same mutation. In all cases they found the same structure which they name closed gate, however given that it is always the same they determined and discussed it for patient 1 while they fully ignore it in the other two patients. Moreover, judging from the figures shown in the manuscript it seems to me that the closed gate structure is the highest quality 2D class also in patient 2 and 3. Even though this closed gate structure is always present, they barely mention it and never show it in the figures.*

Response: We appreciate the reviewer's comment and acknowledge the lack of clarity in this regard. The three patients present fibrils with closed gate folds, which were identified during 3D classification. We did not refine those density maps because of their similarities to the map resolved for Patient 1. We have now included the following text to emphasize that all three patients have fibrils with closed gate folds in conjunction with a second fibril fold (extended, broken, or absent).

For Patient 2, the results heading, page 4, line 43 now reads "Polymorphism of fibrils in ATTRv-I84S patient 2." The results for patient 2 now reads "One 3D class resembled the closed gate fold resolved from patient 1; therefore, we did not continue processing this class further for atomic modelling (Fig. 2b)." on pages 4-5, Lines 45-1.

For Patient 3, the heading in the results for patient 3 is now "**Polymorphism of fibrils in ATTRv-I84S patient 3**" on page 5, Line 40, and the text reads ".Similar to the other two patients, the first class resembled the closed gate fold, and we did not continue processing for atomic modeling (Fig. 3b)" on page 5, lines 42-43.

Perhaps this duality is associated with the presence of both wild-type and mutant proteins since they are heterozygous, as discussed in the discussion section under the heading "Implications in the mechanism of transthyretin aggregation in ATTR amyloidosis", which reads, "We also observe that all three heterozygous ATTRv-I84S patients present with fibrils adopting a closed gate fold, in addition to their unique folds. We speculate that the prevalence of this population of closed gate fibrils may be influenced by the content of wild type transthyretin. Nevertheless, a common core structure that includes a fragment from the N-terminus (from residues Leu 12 to Lys 35) and the C-terminus (from residues Gly 67 to Val 122) in all fibril folds may serve an important role for amyloid formation. The co-existence of different fibril folds within the same fibril also suggests that the common core in the structure may be sufficient to promote elongation and seeding of the different folds" on page 11, lines 27-33.

Reviewer 2, comment 2. *In their other structures, about 80-90% of the residues form the usual TTR amyloid fold which is common to the closed gate structure and to all other TTR structures so far reported. The polypeptide stretch they find in different conformations is a solvent exposed polypeptide segment which in some case is found in different conformations and sometimes is simply invisible (maybe it is simply proteolysed?). In any case let's keep in mind that structural biology is full of examples showing that even two structures from the very same sample maynot be identical, this may be especially true for ex-vivo structures where different part of the tissue may have slightly different physio-pathological conditions and amyloid deposits indifferent patients may have significantly different age.*

Response: We thank the reviewer for their input. We agree that the same protein can lead to different structures in vivo, and there are many studies reflecting this, including ours [2–8]. To reduce random variability, we intentionally studied three patients that had same ATTR genotypes (they were within the same kindred) with a clinically homogeneous disease (ATTRv-I84S). Additionally, the samples were all extracted from the left ventricle of autopsied hearts and patients were in the same age range at the time of collection. Thus, we believe that the structural variability observed in the fibrils from ATTRv-I84S patients is relevant to the mutation.

Reviewer 2, comment 3. *In summary, the authors overinterpret these minor differences as to claim that different patients have different structures and even challenge the idea of one disease associates with one fibril fold proposed after the structural data obtained on A-beta and tau fibrils. These claims are baseless as the structural data point exactly in the opposite direction. Structural data instead show that the I84S mutation partially destabilises the closed gate conformation leading to a mixture of conformers, but it also shows the resilience of TTR amyloid fold. Thus, the title, abstract, presentation of the data, discussion and take-home message must be completely revised and made consistent with the experimental data.*

Response: We appreciate the reviewer's concern. Our data points to conformational changes and the existence of fibril polymorphs in ATTRv-I84S amyloidosis patients, suggesting that "Our finding suggests that one amyloid disease may associate with multiple fibril folds in

systemic amyloidoses, calling for further studies.", as the Abstract now reads on page 1, lines 36-37. Our intention was not to dismiss years of work on brain amyloid diseases, and we regret that it came across as such. Considering the reviewer's suggestion, we have modified the text in our revised manuscript. On page 10, lines 4-18, the discussion reads, "A recent study reveals that mutant a-synuclein fibrils from one individual suffering from early-onset Parkinsonism (known as Juvenile Onset Synucleinopathy), depict a distinct fold that differs from the folds of wild-type a-synuclein[29]. In our study, although the ATTR fibrils were obtained from the same organ, the heart, from patients with the same disease-associated mutation, ATTRv-I84S, they adopt various folds that are individual specific.

It may be possible that this inconsistency may be attributed to the systemic nature of ATTR amyloidosis. Fibril polymorphism in systemic diseases has been previously investigated in an independent study using light chain fibrils from an AL amyloidosis patient[34]. Similar to what we observe, different AL fibril structures can coexist within the same fibril in the same individual. On the other hand, serum amyloid A (SAA) fibrils from two AA amyloidosis patients show distinct structures when obtained from vascular and glomerular regions of the kidney, and the involvement of the two regions is associated with distinct clinical phenotypes[35,36]. Overall, the structural landscape of fibrils in systemic amyloidosis may be diverse and requires further studies on each individual disease. Based on our observations, we speculate that fibril polymorphism in ATTR amyloidosis, when it occurs within the same disease and the same organ, may be individual specific and driven by the presence of the mutation."

The conclusion reads "Our study provides further evidence that one disease may associate with multiple fibril folds in systemic amyloidoses." page 11-12, lines 45-1.

Reviewer 2, comment 4. *The estimated energy of salvation shows that all fibrils are based on the same stabilising core with the channel giving a minor contribution. That's why the channel region can be found indifferent conformations.*

Response: We thank the reviewer for pointing this out. We have added this to the discussion in paragraph 1 "The estimated energy of solvation shows that in all fibril polymorphs from our study, the core contributes predominantly to the stability of the fibrils with the channel providing only a minor contribution, perhaps explaining why the channel can be found in different conformations." on page 11, lines 4-7.

Reviewer 2, comment 5. *Fig 5 and Fig S8 are misleading, the closed structure is present in all three patients but the authors are not showing them here or elsewhere. It is crucial to mention and show them allover.*

Response: We appreciate the reviewer's concern regarding the representation of closed fold. We have made changes to both Figures 5 and Supplementary Fig. 9. We have simplified this panel to represent all folds that we see.

<u>Reviewer 2, comment 6.</u> *pg 13 contrast to one fibril one disease: it is highly speculative, this polymorphs show simply that the I84S destabilise the internal gate of TTR fibrils. Moreover the seem to forget here that they have the same closed conformation in all patients.*

Response: The abstract now reads "Our finding suggests that one amyloid disease may associate with multiple fibril folds in systemic amyloidoses, calling for further studies." on page 1, lines 36-37.

The conclusion is now reading "In summary, our cryo-EM study demonstrates the existence of polymorphism in ATTRv-I84S fibril structures. Our study provides further evidence that one disease may associate with multiple fibril folds in systemic amyloidoses. We also discuss a possible mechanism through which mutations affect ATTR fibril polymorphism, by driving structural changes in the polar pocket, which do not reduce their extraordinary stability. Overall, our study opens questions about the structural implications of mutations in ATTR amyloidosis and other amyloid conditions." on page 11-12, lines 44-4.

With regards to the Closed Gate fold, we have addressed the reviewers' concerns (See above, Reviewer 2 Comment 1).

<u>Reviewer 2, comment 7.</u> *paragraph pg 14 is very speculative: if the different conformations seen in patients are affected or affect proteolysis, this should be checked by MS of fibrils.*

Response: In response to the reviewer's comment, we performed mass spectrometry analysis of the three patient samples (Supplementary Table 1 and 2, and supplementary fig. 3). We found that fibril samples from all patients have similar compositions, suggesting that proteolysis may not influence the formation of these new polymorphs. We have included Supplementary Table 1 and 2, and supplementary fig 3 (see above, Reviewer #1, comment 3) and the text in the discussion section under the heading "Mass spectrometry of the ATTRv-I84S fibrils in our study show that all three patient samples have similar fragment composition (Supplementary Table 1 and 2, and Supplementary Fig. 3), including near the polar pocket region. This finding and the strategic location of the I84S mutation (Fig 1i) suggests that rather than differential proteolytic events, the I84S mutation drives the observed structural polymorphism." on page 10, lines 27-31.

For figure and tables, see above, reviewer 1 comment 3.

<u>Reviewer 2, comment 8</u>. *The claim that the content of the cavity may play a role in pathology is way too speculative*

Response: Studies suggest that cavities can serve as ligand binding pockets that contribute to pathogenesis in amyloidosis. These studies include tau fibrils from chronic traumatic

encephalopathy (CTE) patients. Our speculations on the role of cavities are drawn from these studies and our observations. In response to the reviewer, we have incorporated these references into the manuscript in the discussion section under the title "Implications of the polar pocket in pathology", as reads: "Perhaps, the content of the channel or pocket could play a role in pathology as speculated in the case of neurodegenerative diseases[41]. Many other fibril structures contain polar or hydrophobic channels with unknown functions too[28]." on page 11, lines 9-11.

Reviewer 2, comment 9: *electrophoretic and MS analysis of the fibrils would be very useful to provide explanations to the different conformation found in the three patients.*

Response: In response to the reviewer, we performed mass spectrometry analysis for fibrils from all three patients. We observe that fibril samples from all three patients have similar composition (Supplementary Table 1 and 2 and Supplementary Fig. 3). Based on this evidence and the strategic location of the I84S mutation in the fibril structure, we suggest that, rather than differential proteolytic events, the mutation drives the observed structural polymorphism. Now the text reads as: "Mass spectrometry of the ATTRv-I84S fibrils in our study show that all three patient samples have similar fragment composition (Supplementary Table 1 and 2, and Supplementary Fig. 3), including near the polar pocket region. This finding and the strategic location of the I84S mutation (Fig 1i) suggests that rather than differential proteolytic events, the I84S mutation drives the observed structural polymorphism." on page 10, lines 27-31.
For figure and tables, see above, reviewer 1 comment 3.

Reviewer 2, comment 10: *It is very burdensome to have supplementary figures embedded in the main text, please remove the supplementary figures from the main text.*

Response: We thank the reviewer for pointing this out. We have separated the supplementary figures from the main text and figures.

Reviewer 2, comment 11: *supplementary figure 3 the percentages are wrong as they do not take in account the particles which are not used for 3D reconstruction*

Response: As suggested, we have removed the percentage of particles from the figure. Check the modified supplementary figure 4.

Reviewer 2, comment 12: *page 10 correct "mayor" with "major"*

Response: Thank you. We corrected the typological error.

Reviewer 2, comment 13: *pg 10 the missing region in the fibrils is not folded as coiled coil in the native structure but it is a beta strand - loop - beta edge strand.*

Response: Thank you, we have made the corrections. Now the text reads as "Residues Ala 36 to His 56, missing in the structure of ATTR fibrils, are located on the outside of the tetrameric protein and contained both structured (β-strands) and unstructured (loop) regions surrounding strands C and D (Supplementary Fig. 10, and Figs. 4b-c). The residues involving the gate (Leu 57 to Gly 67) of the ATTR fibrils are also located on the outside of the tetrameric protein and are unstructured (Supplementary Fig. 10, and Fig. 4b-c)." on page 8, lines 18-27.

Reviewer 2, comment 14: *when rmsd is calculated it is never stated on how many Ca*

Response: Thanks for pointing out. We have modified the text and added the "Cα" to the Fig. 4a caption.

Reviewer 2, comment 15: *Fig. 1E and H, 2E and 3E: the resolution range goes to 5.6 Å but the resolution seems to be very homogeneous, my best guess is that there is a problem in the calculation of the resolution or else the color range should modified to appreciate the local differences in resolution.*

Response: The resolution range is kept up to 5.6 Å to compare the local resolution differences between all structures, thus the local resolution range is adjusted to the same scale. In response to the reviewer, we have now also added the figures with individual resolution ranges separately as Supplementary Fig. 5. The text is also updated at the end of the discussion for patient 3, as follows: "For additional structural reference, we have included the maps of all four structures obtained in this study and their local resolution in Supplementary Fig. 5.

Reviewer #3:

Reviewer 3, comment 1: *Regarding the proposal that "this finding challenges the paradigm of one amyloid disease associates with one fibril fold. I recall Strohaker, T., et al. (2019). "Structural heterogeneity ofalpha-synuclein fibrils amplified from patient brain extracts." Nat Commun 10(1): 5535, in which the authors raised questions regarding the one disease-one strain hypothesis. Similar questions have been raised in several papers [https://doi.org/10.1038/s41586-020-2317-6;DOI: 10.1002/anie.201511524; https://doi.org/10.1038/s41467-019-13564-w]. The authors should clarify the uniqueness of this paper in comparison with those related papers.*

Response: We thank the reviewer for sharing these papers. Strohäker, et al.[9] discuss amplified or seeded recombinant fibrils, which are shown to lead to polymorphic structures that may differ from those found *in vivo* [10,11]. Annamalai et al[12]. show the existence of polymorphism in AL ex vivo fibrils. However, they do observe these differences based on negative stain electron microscopy which does not provide a "molecular level" detail provided by cryo-EM. Schweighauser et al.[13] show the existence of two fibril types in the brains of 5 MSA patients, which

were found to be different from those of DLB patients. All these studies targeted fibrils from brain amyloid diseases. Compared to these papers, our work focuses on ex vivo systemic ATTR amyloid fibrils resolved with cryo-EM. Our paper demonstrates that in contrast to localized amyloidosis, systemic ATTR fibrils do not follow the *one disease, one fold* paradigm. Consistent with the paper by Radamaker et al. (2021, Nat Comm)[14] showing the presence of different morphologies in light chain fibrils, our study provides further evidence that this phenomenon can occur in multiple patients of systemic ATTR amyloidosis. We have included this relevant reference in the revised manuscript as follows (page 10, lines 10-12) "Fibril polymorphism in systemic diseases has been previously investigated in an independent study using light chain fibrils from an AL amyloidosis patient[34]. Similar to what we observe, different AL fibril structures can coexist within the same fibril in the same individual."

<u>Reviewer 3, comment 2:</u> *Figure 6. A schematic representation of the fibril polymorphism observed in ATTR fibrils: Although polymorphs shown in Fig. 6 are distinct in detail, they are similar to each other in the core structures. Recently, Wilkinson et al. (https://doi.org/10.1038/s41467-023-36791-8)reported a paper showing that a hammer-shaped motif makes up the core of all b2-microgobluin polymorphs. It seems that a common core structure is an important observation for understanding the mechanism of TTR amyloid formation, as well as polymorphs observed in non-core regions.*

Response: We thank the reviewer for their insights. We have included this in the discussion section under "Implications in the mechanism of transthyretin aggregation in ATTR amyloidosis" as follows: "Nevertheless, a common core structure that includes a fragment from the N-terminus (from residues Leu 12 to Lys 35) and the C-terminus (from residues Gly 67 to Val 122) in all fibril folds may serve an important role for amyloid formation." on page 11, lines 29-31.

<u>Reviewer 3, comment 3:</u> *Sup. Fig. 2: "The analyzed ATTRv-I84S patients are type A with intact TTR and C-terminus TTR fragment". Could the authors specify the residue numbers for the C-terminus fragment? Is that TTR50-127? From Sup. Fig. 2C lane 3, the fraction of the C-terminus fragment is higher than 50% of the total. For lane 3, the bands for the intact and fragment TTR are unclear. All CryoEM structures reported in this paper show the entire regions although N-terminal (1-10) and 35-55 regions were missing (Fig. 4). Then, how do the intact and fragmented TTRs coexist in fibril structures determined in this paper?*

Response: We have both intact and fragmented TTR in the fibril samples, as seen by western blot (Supplementary Fig. 2c). We evaluated the presence of C-terminal fragments by western blotting using an antibody that specifically recognizes these fragments. The Type B patient that we use as a control contains only full-length transthyretin in the fibril extracts, whereas the three extracts from ATTRv-I84S patients present a band corresponding to C-terminal fragments. This has been reported as Type A amyloid fibrils[15]. We envision that certain layers of the cryo-EM fibril structures comprise of the full length TTR and some layers have fragments. Due to the disordered nature of the region 35-55 in the cryo-EM structure, they may not be visible.

Reviewer 3, comment 4: *In type A fibrils, when does the proteolytic cleavage occur? Is that after formation of amyloid fibrils, leaving the cleaved peptides in fibrils and making the structural analysis with the nicked N-terminal peptide possible? Probably, this is not the case.*

Response: Based on the previous study on V30M from Schmidt et al[16] and our own observations, cleavage likely occurs after the fibril formation. The nicked N terminal is held in the structure by stacking interactions between the layers of the fibril and through the side chain interactions with the C-terminal interface. This information is now included in the Discussion section under the heading "**Implications in the mechanism of transthyretin aggregation in ATTR amyloidosis"** as follows: "Our mass spectrometry analysis and structures suggest that, similar to previously described ATTR structures, ATTRv-I84S fibrils are compatible with a proposed proteolytic mechanism in where proteolysis occurs after fibril formation[21,22] The presence of two individual N- and C- fragments encompassing the core of the ATTR fibril structures at a 1:1 ratio makes it unlikely for proteolytic event to happen before fibril formation." on page 11, lines 16-20.

Reviewer 3, comment 5:. *Page 14 "The structural variability that we found in cardiac ATTR fibrils indicates that type A pathology can consist of structural polymorphs." Is it possible that proteolytic cleavage affects the CryoEM analysis leading to apparent structural polymorphs?*

Response: Following the reviewer's suggestion, we performed additional mass spectrometry studies of ATTRv-I84S fibrils. We observe that fibrils from all three patients have a similar fragment composition (Supplementary Table 1 and 2 and Supplementary Fig. 3), thus suggesting that factors other than proteolytic cleavage may be involved in generating these polymorphs. We have added this to the discussion of structural polymorphism in type A in the discussion section, which now reads, "Mass spectrometry of the ATTRv-I84S fibrils in our study show that all three patient samples have similar fragment composition (Supplementary Table 1 and 2, and Supplementary Fig. 3), including near the polar pocket region. This finding and the strategic location of the I84S mutation (Fig 1i) suggests that rather than differential proteolytic events, the I84S mutation drives the observed structural polymorphism." on page 10, lines 28-32.

Reviewer 3, comment 6:. *Fig. 1K. Sup. Fig. 4. "Evidence for the presence of different morphologies within the same fibril". This observation is amazing although the validity should be carefully confirmed. The authors established seeding method for amplifying amyloid fibrils purified from patients (Sup.Fig. 2B). Do the different morphologies within the same fibril propagate during in vitro seeding? Or is it a rare phenomenon only occurring in vivo?*

Response: The presence of different morphologies on the same fibril have been reported for ex vivo fibril structures of immunoglobulin light chain (λ3 LC)[8] and TDP43 (Falcon 2023)[17]. It is therefore possible that this phenomenon exists in other fibrils, consistent with what is observed by us. The investigation of whether these structures are recapitulated in vitro by seeding requires

further and comprehensive cryo-EM studies that are currently in our list of future directions. Based on previous studies[11,18], we anticipate that this endeavor will be challenging. None of these in vitro studies have reported the co-existence of different structure folds within the same fibril, although this may not necessarily mean that it does not occur.
To highlight previous evidence of the co-existence of fibril morphologies within the same ex-vivo fibril, we have included the following text in the revised manuscript (page 10, lines 9-11): "Fibril polymorphism in systemic diseases has been previously investigated in an independent study using light chain fibrils from an AL amyloidosis patient[34]. Similar to what we observe, different AL fibril structures can coexist within the same fibril in the same individual."

Reviewer 3, comment 7:. *Fig. 5. "Fibril stability in ATTRv-I84S amyloidosis fibrils". "ATTR fibrils on average are estimated significantly more stable than other fibrils, both by residue and chain (Figure 5A and5B)". Experimental evidence supporting this argument will be necessary.*

Response: We agree with the reviewer that confirmation of this observation will require experimental validation. Our analysis is an in-silico calculation of the stability, which provides an estimate of the fibril stability, performed similar by others[19,20]. The validation would require the comparison of stability for multiple fibril types, requiring high purity. Homogeneity of the sample is also another limitation for biophysical studies, since contaminants may interfere with relative measurements. We acknowledge that the lack of experimental validation of the stability is a limitation of our study and we have added this to the *Limitation* section in Discussion, as follows: "**Limitations of this study.** Because of our limited access to the clinical histories' of these patients, correlations between disease progression and structure cannot be unequivocally discerned. Additionally, in this present study, we have looked at heart samples from three patients with one mutation. Future studies that involve structural analysis of more mutations and other organs as well as experimental validation of their stability can shed further light on the role of mutations and the tissue microenvironment in structural polymorphism in ATTR amyloidosis." on page 11, lines 37-42.

Rebuttal References:

1. Liepnieks, J. J., Wilson, D. L. & Benson, M. D. Biochemical characterization of vitreous and cardiac amyloid in Ile84Ser transthyretin amyloidosis. *Amyloid Int. J. Exp. Clin. Investig. Off. J. Int. Soc. Amyloidosis* **13**, 170–177 (2006).
2. Falcon, B. *et al.* Structures of filaments from Pick's disease reveal a novel tau protein fold. *Nature* **561**, 137–140 (2018).
3. Falcon, B. *et al.* Novel tau filament fold in chronic traumatic encephalopathy encloses hydrophobic molecules. *Nature* **568**, 420–423 (2019).
4. Fitzpatrick, A. W. P. *et al.* Cryo-EM structures of tau filaments from Alzheimer's disease. *Nature* **547**, 185–190 (2017).
5. Gremer, L. *et al.* Fibril structure of amyloid-β(1-42) by cryo-electron microscopy. *Science* **358**, 116–119 (2017).
6. Guerrero-Ferreira, R. *et al.* Cryo-EM structure of alpha-synuclein fibrils. *Elife* **7**, (2018).

7. Liberta, F. *et al.* Cryo-EM fibril structures from systemic AA amyloidosis reveal the species complementarity of pathological amyloids. *Nat. Commun.* **10**, 1–10 (2019).
8. Radamaker, L. *et al.* Cryo-EM reveals structural breaks in a patient-derived amyloid fibril from systemic AL amyloidosis. *Nat. Commun.* **12**, 1–10 (2021).
9. Strohäker, T. *et al.* Structural heterogeneity of α-synuclein fibrils amplified from patient brain extracts. *Nat. Commun.* **10**, 5535 (2019).
10. Lövestam, S. *et al.* Assembly of recombinant tau into filaments identical to those of Alzheimer's disease and chronic traumatic encephalopathy. *Elife* **11**, e76494 (2022).
11. Li, X. *et al.* Subtle change of fibrillation condition leads to substantial alteration of recombinant Tau fibril structure. *iScience* **25**, 105645 (2022).
12. Annamalai, K. *et al.* Polymorphism of Amyloid Fibrils In Vivo. *Angew. Chem. Int. Ed. Engl.* **55**, 4822–4825 (2016).
13. Schweighauser, M. *et al.* Structures of α-synuclein filaments from multiple system atrophy. *Nature* **585**, 464–469 (2020).
14. Radamaker, L. *et al.* Cryo-EM structure of a light chain-derived amyloid fibril from a patient with systemic AL amyloidosis. *Nat. Commun.* **10**, (2019).
15. Ihse, E., Suhr, O. B., Hellman, U. & Westermark, P. Variation in amount of wild-type transthyretin in different fibril and tissue types in ATTR amyloidosis. *J. Mol. Med. (Berl).* **89**, 171–180 (2011).
16. Schmidt, M. *et al.* Cryo-EM structure of a transthyretin-derived amyloid fibril from a patient with hereditary ATTR amyloidosis. *Nat. Commun.* **10**, 1–9 (2019).
17. Arseni, D. *et al.* TDP-43 forms amyloid filaments with a distinct fold in type A FTLD-TDP. *Nature* **620**, 898–903 (2023).
18. Creekmore, B. C., Chang, Y.-W. & Lee, E. B. The Cryo-EM Effect: Structural Biology of Neurodegenerative Disease Aggregates. *J. Neuropathol. Exp. Neurol.* **80**, 514–529 (2021).
19. Sawaya, M. R. Amyloid Atlas 2023. https://people.mbi.ucla.edu/sawaya/amyloidatlas/ (2023).
20. Sawaya, M. R., Hughes, M. P., Rodriguez, J. A., Riek, R. & Eisenberg, D. S. The expanding amyloid family: Structure, stability, function, and pathogenesis. *Cell* **184**, 4857–4873 (2021).

REVIEWER COMMENTS

Reviewer #1 (Remarks to the Author):

The authors have addressed my previous concerns. I am happy with the changes. I have no further concerns or comments.

Reviewer #2 (Remarks to the Author):

The manuscript “Structural polymorphism of amyloid fibrils in ATTR amyloidosis revealed by cryo-electron microscopy” by Nguyen et al describes the ex vivo structures of the amyloid deposits found in three ATTR patients carrying the same amyloidogenic TTR mutation (I84S).

This revised version has significantly improved compared to the previous one, however incorrect descriptions of the scientific data remain.

Major points:

- Abstract:

“We found that every ATTRv- I84S patient had cardiac fibrils with distinct structures, and these can co-exist within the same fibril. Our finding suggests that one amyloid disease may associate with multiple fibril folds in systemic amyloidoses, calling for further studies.”

“Distinct structures” mean different structures I copied here below one figure as example: these are two of the distinct structures with multiple folds as the Authors define them. Fold indicates the 3D organization of a protein, here it is obviously the same fold where one is lacking two secondary structures.

One more consideration on this topic: by definition, polymers are formed by identical building blocks and amyloids are polymers. Authors say that different polymorphs co-exist in the same fibril, this rules out that each TTR molecule can fold in different ways. See also the introduction paragraph copied below.

- Introduction:

“Moreover, we found that each patient has at least two distinct ATTR fibril structures, and they can co-exist within the same fibril. Our study discusses the source and effects of fibril polymorphism in ATTR amyloidosis and whether there is a potential association between phenotype and fibril structure in systemic amyloidoses.”

“phenotype and fibril structure” what they can discuss is the (minor) structural variability between individuals and the (minor) structural variability between different TTR mutants.

- Fig. 2 it is still comparing the closed structure of patient 1 with the absent gate of patient 2 as if there was no closed structure in patient 2. They did not determine its structure but the structure of the closed gate is obviously present in patient 2. Thus, the message from this figure is clearly misleading. Same holds for figure 3.

- Figure 6: I would strongly suggest adding info on the % of particles in the different conformations found in the patients. Is the closed gate the most common structure in every patient? In any case it is a very important info to add (probably also in figures 1, 2 and 3).

- Discussion (lines 461-464):

"The co-existence of different fibril folds within the same fibril also suggests that the common core in the structure may be sufficient to promote elongation and seeding of the different folds. Overall, our results suggest that the mechanism of aggregation of transthyretin into specific structural folds may be determined by the dissociation of tetramers as well as mutation- induced structural alterations."

This is another example of using "fold" in the wrong contest. In general, all the discussion would hold if the structures were poorly superposable and very different while in this case the different closed/open/broken gate structures are identical except about ten residues that are visible/invisible depending on the classes.

Overall, even if the Authors somewhat toned down their claim of structural diversity between these structures, this manuscript still gives a take-home message which is not based on the presented structural data.

Reviewer #3 (Remarks to the Author):

I checked the revised manuscript and responses to my comments as well as to comments by other reviewers. The authors carefully revised the manuscript considering all the comments. Particularly, they moderated the argument (Abstract): "Our finding suggests that one amyloid disease may associate with multiple fibril folds in systemic amyloidoses, calling for further studies". Now, the paper is important for addressing the role of amyloid polymorphs in disease phenotypes. I am pleased to recommend the publication of the paper.

We are grateful to all the reviewers for their comments and thoughtful analysis of our study. We found the detailed comments and remarks very valuable and constructive.

Point-by-point response to Reviewer 2's comments and suggestions

Reviewer 2, comment 1. *Abstract :"We found that every ATTRv- I84S patient had cardiac fibrils with distinct structures, and these can co-exist within the same fibril. Our finding suggests that one amyloid disease may associate with multiple fibril folds in systemic amyloidoses, calling for further studies." "Distinct structures" mean different structures I copied here below one figure as example: these are two of the distinct structures with multiple folds as the Authors define them. Fold indicates the 3D organization of a protein, here it is obviously the same fold where one is lacking two secondary structures.*

One more consideration on this topic: by definition, polymers are formed by identical building blocks and amyloids are polymers. Authors say that different polymorphs co-exist in the same fibril, this rules out that each TTR molecule can fold in different ways. See also the introduction paragraph copied below.

Response:

We thank the reviewer for their suggestion. We have modified the word "fold" throughout the manuscript to ensure clarity. The abstract now reads, "We found that in each ATTRv-I84S patient, the cardiac fibrils exhibited different local conformations, and these variations can co-exist within the same fibril. Our finding suggests that one amyloid disease may associate with multiple fibril structures in systemic amyloidoses, calling for further studies." (Page 1, Lines 34-37)

As for different structures within the same fibril, the reviewer raises an interesting question. In a study of AL amyloid fibrils, Radamaker et al (*Nature Communications*, 2021) report the majority of fibrils to show a mixture of conformations that switch arbitrarily throughout the fibril length. In addition, a recent paper by Arseni et al. (*Nature*, 2023) demonstrates that in TDP43 amyloid fibrils, different local structural variations could co-exist in individual filaments. Overall, these studies suggest that amyloid filaments do not always adopt identical repetitive structures and are more heteropolymeric than initially thought. Our study is consistent with these previous observations.

Reviewer 2, comment 2. - *Introduction: "Moreover, we found that each patient has at least two distinct ATTR fibril structures, and they can co-exist within the same fibril. Our study discusses the source and effects of fibril polymorphism in ATTR amyloidosis and whether there is a potential association between phenotype and fibril structure in systemic amyloidoses." "phenotype and fibril structure" what they can discuss is the (minor) structural variability between individuals and the (minor) structural variability between different TTR mutants.*

Response:

In response to the reviewer's suggestions, we have modified the Introduction text, which now reads, “Moreover, we found that ATTR fibril structures from each patient exhibited local structural variations, co-existing within the same fibril. Our study discusses the source and effects of fibril polymorphism in ATTR amyloidosis and whether there is a potential association between phenotype and fibril structure in systemic amyloidoses.” (Page 2, Lines 27-30).

Additionally, our discussion now reads,

“Our study demonstrates that despite the consistent phenotype associated with ATTRv-I84S mutation, we observe local polymorphism within and between patients from the same kindred (Fig. 6).” (Page 9, Lines 32-33)

“In our study, although the ATTR fibrils were obtained from the same organ, the heart, from patients with the same disease-associated mutation, ATTRv-I84S, they adopt different structures with local variations that are individual specific.” (Page 10, Lines 7-9).

“This finding and the strategic location of the I84S mutation (Fig 1i) suggests that rather than differential proteolytic events, the I84S mutation drives the observed local structural polymorphism.” (Page 10, Lines 32-33)

“The co-existence of different local conformations within the same fibril also suggests that the common core in the structure may be sufficient to promote elongation and seeding of the different structures. Overall, our results suggest that the mechanism of aggregation of transthyretin into different structures with local variations may be determined by the dissociation of tetramers as well as mutation-induced structural alterations.” (Page 11, Lines 33-37).

“In summary, our cryo-EM study demonstrates the existence of local structural variations in ATTRv-I84S fibrils. Our study provides further evidence that one disease may associate with multiple fibril structures in systemic amyloidoses.” (Page 12, Lines 1-3)

Reviewer 2, comment 3. *Fig. 2 it is still comparing the closed structure of patient 1 with the absent gate of patient 2 as if there was no closed structure in patient 2. They did not determine its structure but the structure of the closed gate is obviously present in patient 2. Thus, the message from this figure is clearly misleading. Same holds for figure 3.*

Response:

To ensure clarity, we have made the following modifications to the results section:

“We performed 3D classification of curvy ATTRv-I84S fibrils from patient 2, yielding three classes that could be visually grouped into two folds (Fig. 2a-b and Supplementary Fig. 4). One fold resembled the closed gate fold resolved from patient 1; we assumed that their structures are the same and therefore, we did not continue processing this class further for atomic modelling (Fig. 2b and Supplementary Fig. 4).” (Page 5, Lines 1-4)

“3D classification of fibrils from patient 3 yielded two classes that could be visually grouped into two distinct folds (Fig. 3a-b and Supplementary Fig. 4). Similar to the other two patients, the first class resembled the closed gate fold, and e did not continue processing for atomic modeling (Fig. 3b and Supplementary Fig. 4). Therefore, we utilized the closed gate fold model obtained from patient 1 for comparison purposes.” (Pages 4-5, Lines 44-2)

<u>Reviewer 2, comment 4</u> *Figure 6: I would strongly suggest adding info on the % of particles in the different conformations found in the patients. Is the closed gate the most common structure in every patient? In any case it is a very important info to add (probably also in figures 1, 2 and 3).*

Response:

On the first revision of our paper, Reviewer #2 commented: “supplementary figure 3 the percentages are wrong as they do not take in account the particles which are not used for 3D reconstruction”

We agree with this comment because not all filaments were selected for data processing and only the best particles were used for 3D reconstruction. So, the percentages are somewhat biased and likely meaningless. Consequently, and after deep discussion, I and the rest of the authors have decided not to include it and thus avoid misinterpretations. However, we include those numbers here for the reviewers’ perusal.

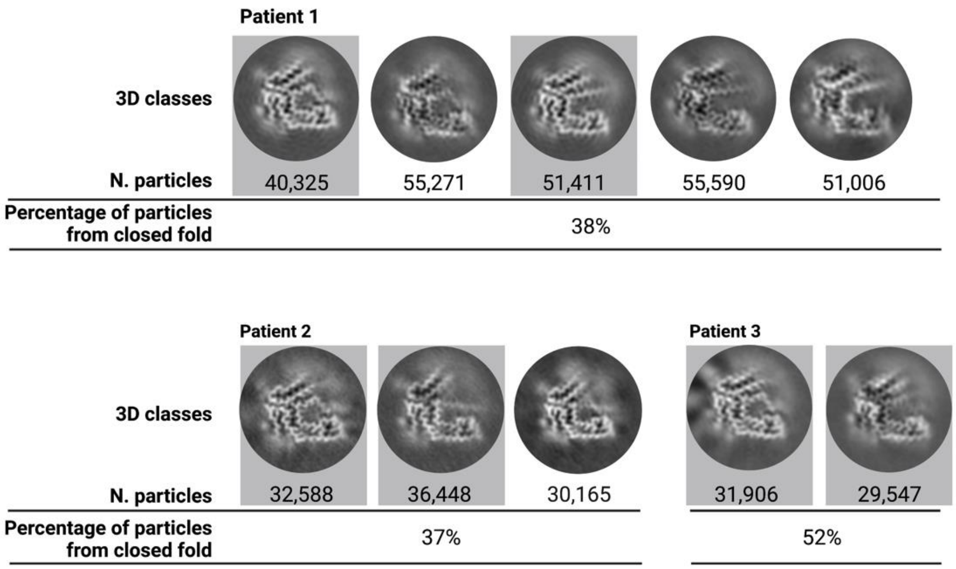

Rebuttal Figure 1. 3D classification of fibril particles resulted in various distinct particle populations in each of the patients. The percentage of particles belonging to each fold is shown here. Shaded maps are representative maps used in this paper.

<u>Reviewer 2, comment 5.</u> *“The co-existence of different fibrilfolds within the same fibril also suggests that the common core in the structure may be sufficient to promote elongation and seeding of the different folds. Overall, our results suggest that the mechanism of aggregation of transthyretin into specific structural folds may be determined by the dissociation of tetramers as well as mutation- induced structural alterations.”*

This is another example of using “fold” in the wrong contest. In general, all the discussion would hold if the structures were poorly superposable and very different while in this case the different closed/open/broken gate structures are identical except about ten residues that are visible/invisible depending on the classes.

Response:

In response to the reviewer's suggestion, we have modified the text which now reads, **“**The co-existence of different local conformations within the same fibril also suggests that the common core in the structure may be sufficient to promote elongation and seeding of the different structures. Overall, our results suggest that the mechanism of aggregation of transthyretin into different structures with local variations may be determined by the dissociation of tetramers as well as mutation-induced structural alterations.” (Page 11, Lines 33-37)

References

Arseni, D., Chen, R., Murzin, A.G. et al. TDP-43 forms amyloid filaments with a distinct fold in type A FTLD-TDP. *Nature* 620, 898–903 (2023). https://doi.org/10.1038/s41586-023-06405-w

Radamaker, L., Baur, J., Huhn, S. et al. Cryo-EM reveals structural breaks in a patient-derived amyloid fibril from systemic AL amyloidosis. *Nat Commun* 12, 875 (2021). https://doi.org/10.1038/s41467-021-21126-2
